# Flavonoid-attracted *Aeromonas* sp. from the Arabidopsis root microbiome enhances plant dehydration resistance

**DOI:** 10.1038/s41396-022-01288-7

**Published:** 2022-07-16

**Authors:** Danxia He, Sunil K. Singh, Li Peng, Richa Kaushal, Juan I. Vílchez, Chuyang Shao, Xiaoxuan Wu, Shuai Zheng, Rafael J. L. Morcillo, Paul W. Paré, Huiming Zhang

**Affiliations:** 1 Shanghai Center for Plant Stress Biology, Center for Excellence in Molecular Plant Sciences, Chinese Academy of Sciences, Shanghai, 201602 China; 2 University of Chinese Academy of Sciences, 100049 Beijing, China; 3 Department of Chemistry and Biochemistry, Texas Tech University, Lubbock, TX 79409 USA; 4 Present Address: Department of Applied Sciences and Biotechnology, Shoolini University, Solan, India; 5 Present Address: Instituto de Tecnologia Química e Biológica (ITQB), Oeiras, Lisbon, Portugal; 6 Present Address: Instituto de Hortofruticultura Subtropical y Mediterránea “La Mayora” (IHSM-UMA-CSIC), Málaga, Spain

**Keywords:** Applied microbiology, Plant sciences

## Abstract

Flavonoids are stress-inducible metabolites important for plant-microbe interactions. In contrast to their well-known function in initiating rhizobia nodulation in legumes, little is known about whether and how flavonoids may contribute to plant stress resistance through affecting non-nodulating bacteria. Here we show that flavonoids broadly contribute to the diversity of the Arabidopsis root microbiome and preferentially attract *Aeromonadaceae*, which included a cultivable *Aeromonas* sp. H1 that displayed flavonoid-induced chemotaxis with transcriptional enhancement of flagellum biogenesis and suppression of fumarate reduction for smooth swims. Strain H1 showed multiple plant-beneficial traits and enhanced plant dehydration resistance, which required flavonoids but not through a sudden “cry-for-help” upon stress. Strain H1 boosted dehydration-induced H_2_O_2_ accumulation in guard cells and stomatal closure, concomitant with synergistic induction of jasmonic acid-related regulators of plant dehydration resistance. These findings revealed a key role of flavonoids, and the underlying mechanism, in mediating plant-microbiome interactions including the bacteria-enhanced plant dehydration resistance.

## Introduction

The root microbiome is critical for the fitness of plants [1–3]. To harness the soil microbiome for plant benefits, it is crucial and challenging to delineate the causal relation between plant metabolites and the microbiome assembly, especially the microbial members that develop mutualistic association with the plant [4–7]. Flavonoids are polyphenolic compounds produced in plants as secondary metabolites, which account for a significant part of root exudates [8–10]. In contrast to the well-known function of some flavonoids as key inducers of rhizobia nodulation in legumes, much remains unclear whether and how flavonoids may affect non-nodulating bacteria in the root microbiome associated with non-legume plants [8–10].

Being sessile, plants under biotic stress may protect themselves through an action hypothesized as “cry-for-help”, i.e., secreting certain diffusible metabolites to attract allies such as parasitic wasps or beneficial soil microbes for better defense against herbivores or pathogens [11–13]. It is unclear whether the cry-for-help strategy is also effective for plants to combat abiotic stress. Flavonoid deficiency in *Arabidopsis thaliana* mutants was reported to cause no significant impacts on root association with a synthetic bacteria community in an aqueous system [14]. In contrast, the potential impacts of elevated flavonoid production on the root microbiome remain elusive. Flavonoid production is often elevated in plants under abiotic stress [15], such as dehydration that is a major threat to global agriculture; meanwhile, natural soil harbors beneficial rhizobacteria that are promising in enhancing plant resistance to dehydration [16–18]. Thus it is intriguing whether and how flavonoid-mediated plant-microbe interactions may improve the resistance of soil-grown plants to dehydration stress.

In this study, we hypothesized that elevated flavonoid production would reshape root microbiome, which might offer cultivable beneficial bacteria that is attracted by flavonoids to help increase plant resistance to dehydration stress through cry-for-help. Our results revealed strong effects caused by elevated flavonoid production on Arabidopsis root microbiome in a natural soil. Analyses of the bacteria community at the family level highlighted *Aeromonadaceae* as the most obviously flavonoid-responsive family, from which a cultivable *Aeromonas* sp. was obtained. Subsequent investigations on this *Aeromonas* sp. revealed that flavonoids induced chemotaxis with transcriptional enhancement of flagellum biogenesis and suppression of fumarate reduction for smooth swims. The *Aeromonas* sp. was identified as a plant-beneficial bacterial strain capable of enhancing plant resistance to dehydration. However, our results indicated that the stress-induced increases in flavonoids contribute to the mutualism by alleviating the negative impacts of the dehydration stress on the pre-established plant-rhizobacteria association, instead of a sudden cry-for-help upon the stress. Further investigations revealed that the bacteria-enhanced plant dehydration resistance results from bacterial regulation of stomatal closure in concomitant with strong synergistic induction of *in planta* regulators of plant dehydration resistances.

## Materials and methods

### Bacteria growth and inoculum preparation

In addition to the newly isolated *Aeromanas* sp. H1, the other bacterial strains used in this study included *B. amyloliquefaciens* GB03 [4], *B. megaterium* YCR-R4 [5], *B. megaterium* TG1-E1 [19]. In addition to the long-term stocks at −80 °C, bacterial strains were streaked on Luria–Bertani (LB) plates (1% NaCl, 1.5% agar) and kept at 4 °C, and were refreshed monthly. To make the inoculum, bacterial strains were cultured in liquid LB medium (30 °C; 220 rpm) to reach the exponential growth phase, as estimated by an optical density measured at a wavelength of 600 nm (OD600) approximately equal to 0.8–1.1. The bacteria cultures were centrifuged at 4000 rpm in an Avanti J-E High-Speed Centrifuge (Beckman Coulter) for 20 min and re-suspended in 0.45% NaCl saline solution for soil inoculation.

### Plant materials and growth conditions

The Arabidopsis mutants *pap1-D* (CS3884) and *tt4-4* (CS66120) were ordered from NASC (The Nottingham Arabidopsis Stock Centre). Seeds were sterilized with 30% house bleach for 10 min. After washing in sterile double-distilled water five times, the seeds were planted on half-strength Murashige and Skoog (MS) medium in Petri dishes. After stratification at 4 °C for 48 h, the seeds were placed in a Percival CU36L5 growth chamber under the following conditions: 22 °C; 16 h day/8 h night light cycle, and ~100 μmol m^−2^ s^−1^ light intensity. After being transferred for soil tests, *Arabidopsis* seedlings were kept in a growth room with a 16 h/8 h day/night cycle at 22 °C for normal growth or 28 °C for dehydration stress experiments. Coriander (*Coriandrum sativum* L.) germinated and grew in soil under the same conditions as the soil-grown Arabidopsis. The japonica rice cultivar K59 [20] germinated and grew in soil in a growth room with 16/8 h light/dark cycle at 28 °C for both normal growth and dehydration stress experiments.

The natural soil substrates were collected from the Chenshan Botanical Garden, Shanghai, China. The soil was cleaned from plant parts, worms and stones and homogenized manually using a sieve (2.5 mm^2^). The cleaned and homogenized field soil was mixed with the commercial soil (Pindstrup Substrate) in 1:1 ratio, in order to avoid the soil being too sticky when it was wet and to avoid forming clogs. The mixed soil was then homogenized again and distributed to each pot. Samples from three pots (18 plants) were collected as one biological replicate. Four biological replicates were grown for each plant genotype.

### Microbiome sample preparation and 16S rRNA gene sequencing

The microbiome DNA from the soil, rhizosphere and endosphere compartments were prepared as previously described [21]. Soil 1 is the initial bulk soil, which was collected at the time of transferring soil into the pots. Soil 2 is the soil from unplanted pots which were subjected to the same conditions as the planted pots to prepare the control soil samples at harvest.

DNA sample preparation and 16S rRNA gene sequencing were performed as previously described [21] with minor modifications. Briefly, the total DNA was extracted with the FastDNA SPIN Kit for Soil (MP Biomedicals, Solon, USA) following the manufacturer’s instructions. The amplicon libraries were generated following the protocol of MiSeq platform (Illumina) for 16S rRNA gene metagenomic sequencing library preparation. The PCR primers 799F (5’-AACMGGATTAGATACCCKG-3’) and 1193R (5’-ACGTCATCCCCACCTTCC-3’), which span ~400 bp of the hypervariable regions V5–V7 of the prokaryotic 16S rRNA gene, were extended to 799F-B and 1193R-B by adding bridging sequences (5’-ggagtgagtacggtgtgc-3’ and 5’-gagttggatgctggatgg-3’) at their 5’ ends to better facilitate the second round of PCR [22]. The PCRs were performed with KAPA HiFi HotStart ReadyMix. For each biological replicate, there were three technical replicates. Triplicate reactions of each sample were pooled and a 5 μl aliquot was inspected on a 2% agarose gel. The PCR primers 799F and 1193R produce a mitochondrial product at ~800 bp and a bacterial amplicon at ~400 bp. The bacterial amplicon was extracted from the gel with sharp scalpel and eluted from the agarose using the QIAquick Gel Extraction kit (Qiagen, Hilden, Germany). Following purification and elution in sterilized double-distilled water, the concentration of the amplicon DNA in each sample was determined by using Qubit dsDNA HS Assay Kit on Qubit2.0. The first-round PCR products were further barcoded during the second-round PCR following the protocol of MiSeq platform (Illumina)for 16S rRNA gene metagenomic sequencing library preparation. The 2nd PCR amplification used unique barcode (Supplementary Table S1G) and indexed sequencing adaptor sequences (Supplementary Table S1G). Samples were sequenced at the Genomics Core Facility at PSC.

### Microbiome data analysis

The 16S rRNA gene sequencing data analysis was performed as previously described [23, 24]. Briefly, the quality of reads was checked with fastqc v0.11.7 and the data were preprocessed with trimmomatics v0.39. The processed high-quality data were assembled with FLASH v1.2.11. The subsequent analysis used QIIME v1.9.1. The assembled reads were compared with the reference database SILVA 138.1 using UCHIME algorithm to detect and remove chimera sequences [23]. Operational taxonomic units (OTU) picking was carried out using the *pick_open_reference_otus.py* script in QIIME at 99% sequence identity with SILVA database v.138.1. The annotation of OTUs was performed with Mothur algorithm [24]. After removing OTUs which belong to mitochondria, Chlorophyta, Archaea and Cyanobacteria, we got 9233 OTUs (Supplementary Table S1A). The alpha- and beta-diversity analysis and statistical analysis were performed as previously described [25]. A total of 737 unique genus were obtained (Supplementary Table S1C). Taxonomic genus were normalized via dividing the reads per taxonomy in a sample by the sum of the usable reads in that sample. Pairwise UniFrac distance and principal coordinates analysis were performed by QIIME. The significant differences between samples (*p* value < 0.01) were assessed by *t*-test in R program [26, 27]. All figures were produced by in-house R program.

### Chemotaxis assays

Naringenin, glucose, quercetin and kaempferol (Sigma-Aldrich) were examined for their potential capacities as chemotaxis attractants for *Aeromanas* sp. H1 and *Bacillus megaterium* YC4R4. The chemotaxis assays were performed as previously described [5] with minor modifications. The test was performed based on a Boyden chamber test, according to manufacturer’s protocols of 5 μm Chemotaxis Assay Kit (CBA-105; Cell Biolabs, Inc., San Diego, CA, USA). In total, 100 μM of chemical solutions were prepared in serum-free medium DMEM (0.5% BSA; 2 mM CaCl_2_ and 2 mM MgCl_2_). Mock conditions were prepared with no chemicals. Bacteria cultures were grown as described above. Diluted bacteria solutions with optical density at 0.05 was used. Relative attraction ratio was expressed as relative fluorescence of membrane-detached cells, which were lysed and quantified using CyQUANT GR fluorescent dye. Fluorescence was measured by a 96-well plate reader Varioskan Flash (Thermo Fisher Scientific, Inc., NH, USA) at 480/520 nm. More than four biological replicates were performed per condition, three independent tests were conducted.

### Measurements of biofilm formation

Biofilm formation was measured as previously described [5]. Briefly, bacteria were cultured in liquid LB medium, and 5 ml diluted solution (OD600 = 0.05) was added to a six-well plastic plate containing either 100 µM glucose, 100 µM naringenin/kaemferol/quercetin, or no additional compound as the mock control. After 24 h growing at 30 °C, the liquid contents were removed and the wells were washed with sterile double-distilled water. The wells were then stained with 0.1% crystal violet for 20 min and washed with sterile double-distilled water three times. Biofilm structures were removed from the wells by adding 2 ml 4:1 (vol:vol) ethanol and acetic acid, with gentle agitating till complete dissolution. Optical density values of the resultant solutions were measured at 530 nm in a Varioskan Flash 96-well plate reader (Thermo Fisher Scientific).

### Bacteria and plant RNA-seq and data analysis

For bacteria RNA sequencing, *Aeromanas* sp. H1 single colony was cultured in liquid LB medium with or without 100 μM naringenin for 8 h. For plant RNA sequencing, the soil-grown Arabidopsis shoots were collected at 3 days after the dehydration treatment. Plant RNA and bacteria RNA samples were extracted by using the RNeasy Plant Mini Kit for RNA extraction (Qiagen) and the RNeasy Protect Bacteria Mini Kit (Qiagen), respectively. An aliquot with 1 μg total RNA per sample was used for library preparation with the NEBNext Ultra Directional RNA Library Prep Kit for Illumina (New England Biolabs; E7420L), following the manufacturer’s instructions. All library preparation and subsequent sequencing processes were performed at the Core Facility of Genomics at the Shanghai Center for Plant Stress Biology.

For both the Arabidopsis and Strain H1 RNA-seq, the raw data were preprocessed by using Trimmomatic (version 0.36). Subsequently, the clean reads of plant and bacteria data were mapped to the *Arabidopsis thaliana* reference genome TAIR10 and the *Aeromonas* sp. H1 genome, respectively, by using HISAT with the default parameters. Number of reads that were mapped to each gene was calculated with the htseq-count script in HTSeq. DEGs were identified by using edgeR with the cut-offs of fold-change ≥1.5 (for plant) or 2 (for bacteria) and false discovery rate (FDR) ≤ 0.05. Gene ontology analysis of Arabidopsis was performed by using the BinGO platform (Cytoscape) with *p* value <0.01 as the cutoff. Uniprot was used for gene cluster organization of H1. Three biological replicates of each sample were used to perform the transcriptome analyses.

### Quantitative real-time PCR

*Aeromonas* sp. H1 and *B. megaterium* YC4-R4 RNA samples were extracted by the RNeasy Protect Bacteria Mini Kit (Qiagen). Complementary DNA was synthesized from equal amounts of total RNA (500 ng) from each sample with random primers by using the EasyScript One-Step gDNA Removal and cDNA Synthesis SuperMix (Trans, AE311-02) according to the manufacturer’s instructions. Real-time PCR was carried out using iQ SYBR Green Supermix (Bio-RAD) on a CFX96 real-time PCR detection system (Bio-RAD). The housekeeping gene, *DNA gyrase subunit B (gyrB)*, was used as the internal control for all reactions. Relative gene expression was derived by using 2^−ΔCT^, where ΔCT represents CT of the target gene minus CT of the reference gene *gyrB*. All primers used are in Supplementary Table S4.

### Measurements of various plant-beneficial traits

#### Measurements of bacteria-produced auxin

The levels of indole-3-acetic acid (IAA) were determined as previously described [28]. Briefly, bacteria were cultured in liquid medium (DF minimal salt) supplemented with 5 mM L-tryptophan. After 5 days of incubation at 30 °C with shaking at 180 rpm, the bacteria culture was centrifuged at 6000 × *g* for 10 min, the supernatant was mixed (1:1, v/v) with Salkowski reagent (12 g of FeCl_3_ per liter in 7.9 M H_2_SO_4_) and incubated in dark at room temperature for 30 min. The color intensity was measured at 530 nm by using a Varioskan Flash 96-well plate reader (Thermo Fisher Scientific). Synthetic IAA (20–200 µM) was used to plot the standard curve.

#### Measurements of bacterial siderophores

Bacteria-produced siderophores were measured as previously described [29]. Briefly, the siderophore assay plates were prepared as follows: add 100 ml of MM9 salt solution (15 g KH_2_PO_4_, 25 g NaCl, and 50 g NH_4_Cl in 500 ml of distilled water) to 750 ml of distilled water, then add 32.24 g piperazine-N,N′-bis (2-ethanesulfonic acid) PIPES to adjust the pH to 6.8. Add 15 g agar and autoclave the solution. While the solution is still hot (~50 °C), add 30 ml of sterile 1% Casamino acid solution and 10 ml of sterile 20% glucose solution, then slowly add 100 ml blue dye solution with agitation and pour the solution into plates. The blue dye solution is prepared and autoclaved in advance by mixing 9 ml of ferric iron solution (0.0027 g of FeCl_3_-6 H_2_O in 10 ml of 10 mM HCl) with 50 ml CAS solution (0.06 g of casamino acid (CAS) in 50 ml of distilled water) and 40 ml HDTMA solution (0.073 g of hexadecyltrimethylammonium bromide (HDTMA) in 40 ml of distilled water). Ten μl of bacteria culture (OD = 0.001) was dropped on the plates and incubated at 30 °C for 3–4 days. Production of siderophore was visualized by an orange color in the form of a halo zone surrounding the bacteria colonies. The siderophore production efficiency is calculated as produced efficiency (S.E) = (S − C)/C, where S = siderophore halo zone (mm), C = Colony diameter (mm).

#### Phosphate solubilization assay

Briefly, bacteria cultures were spotted at the center of plates containing Pikovskaya’s agar medium, which consisted of the following reagents (g/l), 0.5 g yeast extract, 10 g dextrose, 5.0 g Ca_3_(PO_4_)_2_, 0.5 g (NH_4_)_2_SO_4_, 0.2 g KCl, 0.1 g MgSO_4_.7H_2_O, 0.0001 g MnSO_4_, 0.0001 g FeSO_4_.7H_2_O and 15 g agar. The plates were incubated at 30 °C for 5 days. Phosphate solubilization was visualized by a halo zone surrounding the bacteria colony. The phosphate solubilizing efficiency is calculated as Solubilizing efficiency (S.E) = (Z − C)/C, where Z = Solubilization zone (mm), C = Colony diameter (mm).

#### Measurements of bacterial ACC deaminase activities

Briefly, 10 µl of fresh bacteria culture (OD = 0.001) was dropped on DF minimal salt agar media, which contained the following components (g/l), 4.0 g KH_2_PO_4_, 6.0 g Na_2_HPO_4_, 0.2 g MgSO_4_.7H_2_O, 1 mg FeSO_4_.7H_2_O, 10 µg H_3_BO_3_, 10 µg MnSO_4_, 70 µg ZnSO_4_, 50 µg CuSO_4_, 10 µg MoO_3_, 2 g glucose, 2 g gluconic acid, and 2 g citric acid. The medium also contained 3 mM filter-sterilized ACC. Plates were incubated 30 °C for 72 h. Strains that were able to grow on the media containing ACC were recorded as strains with ACC deaminase activity. The efficiency of ACC deaminase activity was expressed as relative bacteria growth ratio by comparing the final bacterial CFUs to initial bacterial CFUs.

#### Detection of H_2_O_2_ catalase activities

Twenty µl bacteria culture (OD600 = 0.8–1) was dropped onto a glass slide. Subsequently 20 µl 30% H_2_O_2_ was added directly to the bacteria culture on the slide. Within 30 s, the droplet would show the effervescence morphology, which is indicative for bacterial H_2_O_2_ catalase activity [30].

#### Measurements of bacteria-produced polyamines

The production of polyamines was measured as previously described [31] with minor modifications. Briefly, bacterial strains were cultured on long ashton decarboxylase medium, which contained (g/l) 0.16 g NH_4_NO_3_, 0.021 g Na_2_HPO_4_·12H_2_O, 0.111 g CaCl_2_·2H_2_O, 0.174 g K_2_SO_4_, 0.185 g MgSO_4_·7H_2_O, 0.4 mg H_3_BO_3_, 0.2 mg MnSO_4_·4H_2_O, 0.03 mg ZnSO_4_·7H_2_O, 0.04 mg CuSO_4_·5H_2_O, 0.003 mg NaMoO_4_·2H_2_O, 0.003 mg CoSO_4_·7H_2_O, 0.021 g FeNaEDTA, 10.0 g glucose, 2.0 g L-arginine monohydrochloride, and 0.02 g phenol red. The plates were incubated at 30 °C for 4 days. Red halos on the yellow background indicated arginine decarboxylation by the bacteria. The quantification polyamines production was calculated as relative produced efficiency (S.E) = R/C, where R = red hole diameter (mm), C = Colony diameter (mm).

### Bacterial enhancement of plant dehydration resistance

Tyndalized soil were prepared as previously described [5]. Seven-day-old Arabidopsis seedlings were transferred to the soil. After 2 days of adaptation, the plants were inoculated by adding 50 ml freshly cultured Strain H1 inoculum (OD600 = 1.0) per soil pot. The same volume of bacteria-free 0.45% NaCl saline solution was used for the mock control. At 2 days post Strain H1 inoculation, the plants were moved from 22 to 28 °C for dehydration treatment by withholding watering. Rice and coriander plants were inoculated with Strain H1 at 10 and 17 days after germination, respectively. The dehydration stress treatment started immediately after H1 inoculation. Soil water content was measured by Moisture Meter Delta-T Devices (HH2). Photosynthesis efficiency was recorded with FluorPen (FP100). Images of the plants were taken typically at ~12 days after the dehydration treatment started. The plants were re-watered shortly after taking the pictures. Plant survival rates were recorded at 2 days after the plants were re-watered.

### Bacteria root colonization measurements

For the in vitro system, 7-day-old seedlings were transferred to vertical plates that contained 1/2-strength MS medium (1% agar) supplemented with the bacteria (OD600 = 0.001–0.002). At 7 days after the transferring, roots were collected and surface-sterilized with 70% ethanol for 20 s, and washed six times with sterilized water. The roots were collected in pre-weighed tubes and fresh weight was recorded. Sterilized 0.45% NaCl was added to the tubes with ten times volume of the root fresh weight (V/W). Each biological replicate contained roots from at least eight seedlings. Samples were machine-homogenized by TissueLyser (Retsch) at a frequency of 25 Hz. A 0.45% NaCl solution was used to prepare serial dilutions for a drop-by-drop seeding on LB agar plates. After overnight culturing at 30 °C, bacteria colonies were counted and expressed as CFU/mg root fresh weight. For in-soil system, roots were harvested at different time points as indicated in the figures. Each biological replicate contained roots from 15 seedlings. The procedure of bacteria quantification was the same as for the in vitro system, except that the roots were surface-sterilized for 10 s.

### Quantification of anthocyanin levels

Anthocyanin levels were determined as previously described [4]. Briefly, shoots and roots of the dehydration-stressed plants were separated and the fresh weight was measured, followed by fine grinding with liquid nitrogen. The extraction buffer (45% methanol, 5% acetic acid) was proportionally added to per unit of fresh weight of the tissues and mixed thoroughly. Two rounds of centrifugation were done to remove the debris, at 12,000 × *g* for 5 min at room temperature. The absorbance of the supernatant was measured at 530 and 637 nm, using a Microplate Reader Thermo Varioskan Flash. 13. Anthocyanin contents (Abs530/g F.W.) were calculated by (Abs530 − (0.25 × Abs657)) × volume added.

### Visualization of air-dry-triggered flavonoids accumulation

Arabidopsis seedlings were vertically grown on 1/2-strength MS medium with 1.1% agar for 7 days. Each plate contained 12 seedlings. The plates had been sealed by porous tapes. Dehydration stress was imposed by putting seedling on microscope slide so as to expose the roots to the air at room temperature for 15 min. Flavonoid accumulation in the roots was stained first with diphenylboric acid 2-amino ethyl ester (DPBA) and visualized by fluorescence excitation at 488 nm with 500–650 nm emission.

### Stomata ROS staining and microscopy

The abaxial epidermal strips of leaves were floated in 0.1 M potassium phosphate buffer (pH = 7.2) for 30 min followed by addition of 2 μM (final concentration) of CM-H_2_DCFDA and further incubated for 20 min at room temperature in dark. After incubation, the strips were washed twice with wash buffer (0.1 mM KCl, 0.1 mM MgCl_2_) for 10 min to remove excess staining buffer. Guard cells in the strips were observed under fluorescent confocal microscope (Olympus DP72 and Leica SP8 Resonant Scanning Confocal). All images were acquired under identical conditions. The fluorescence emission of the guard cells was analyzed using the LAS-AF-2.6.0 equipped with the microscope. ROS levels were indicated by fluorescent signals from the oxidation-sensitive CM-H_2_DCFDA, with the excitation wavelength of 488 nm and the emission wave length at 500–550 nm. The auto-fluorescence signals from chloroplasts were detected with the emission wave length of 670–720 nm.

### Measurements of leaf temperatures

Soil-grown Arabidopsis plants were exposed to the dehydration conditions with or without Strain H1 inoculation as described above. At 4 to 7 days after the treatments, plants in the soil pots were imaged by using a FLIR image camera (FLIR T560-EST). Leaf temperatures were quantified by the software FLIR Tools.

## Results

### Flavonoids broadly contribute to the diversity of Arabidopsis root microbiome

In searching for rhizobacteria that may be favored by elevated flavonoid production, we started by examining Arabidopsis *pap1-D*, a dominant mutant that over-accumulates flavonoids including anthocyanins and flavonols [32, 33]. The plants were grown in natural soil substrates collected from Chenshan Botanical Garden located in Shanghai, China, and 16S rRNA gene sequencing was performed to profile the root microbiome in the three compartments including bulk soil (the soil away from the roots), rhizosphere (the thin layer of soil that is loosely attached to the roots), and endosphere (the surface and the inner parts of the roots) [21]. A total of 1,382,629 effective tags were obtained with an average of 62,847 per sample across all the 22 samples. The effective tags were denoised and the resultant OTUs were subjected to a cutoff with ≥5 reads in all the samples. After removing the OTUs that belong to Mitochondria, Chlorophyta, Archaea and Cyanobacteria, 9233 OTUs were obtained for the subsequent analyses (Fig. S1A and Supplementary Table S1A).

To evaluate the overall impacts of plants on the assembly of microbial communities, we compared the richness of OTUs in bulk soil and the plant-associated microhabitats. Alpha diversity analysis showed that the total numbers of OTUs, including either the observed OTUs or the estimated OTUs obtained by the Chao1 estimator, were much greater in the bulk soil than the endosphere, while the rhizosphere samples showed OTU numbers that were between those of the bulk soil and the endosphere (Fig. S1B, C). Meanwhile, the Shannon index analysis indicated that the community diversity was decreased in the rhizosphere and the endosphere compared to the bulk soil (Fig. S1D). Together the reductions in bacteria richness from the soil to the endosphere reflect the selectivity of plants on root-associated bacteria.

**Fig. 1:**
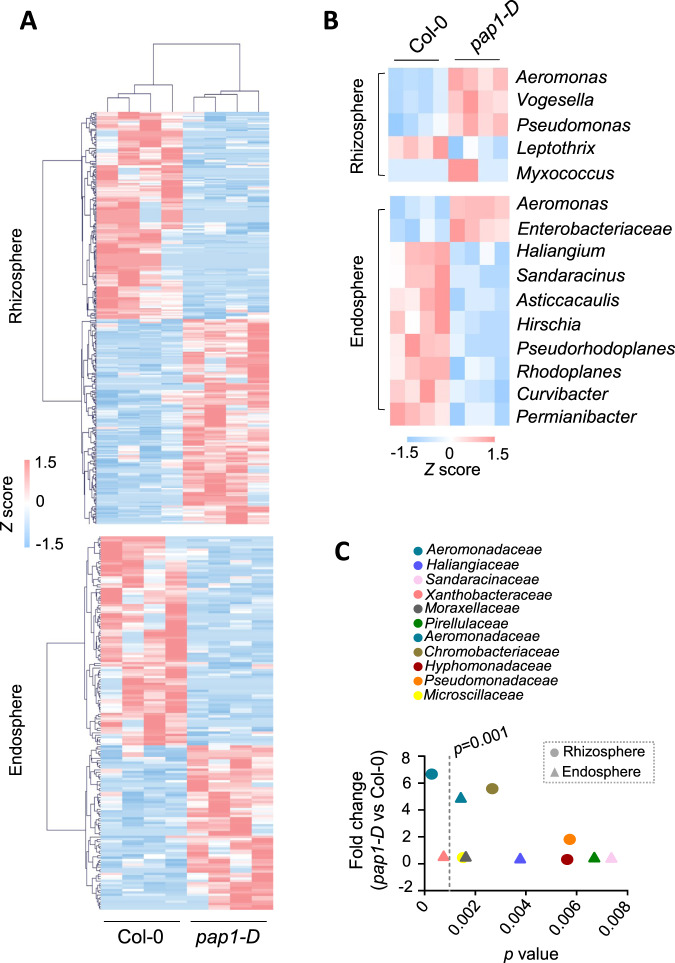
Flavonoids broadly contribute to the diversity of Arabidopsis root microbiome and preferentially attract *Aeromonadaceae*. **A** Heatmaps of the OTUs whose relative abundance (RA) was significantly (*p* < 0.05, *t*-test) altered by the *pap1-D* mutation. **B** The impacts of the *pap1-D* mutation on the root microbiome as evaluated at the genus level. *p* < 0.01, *t-*test. **C** A comparison of the *pap1-D*-affected bacteria families indicates that flavonoids preferentially attract *Aeromonadaceae* in the root microbiome. Each dot represents a mean RA value of 4 biological replicates, each consisting of 18 plants from 3 pots. The *p* values are obtained by using *t*-test in R program.

Compared to the *pap1-D* mutation (i.e., the factor of plant genotype), changes in the factor of compartments had stronger impacts on the microbiome, as shown by principal coordinate analysis of the weighted UniFrac distances between samples (Fig. S2A). Nonetheless, the *pap1-D* mutation resulted in 303 and 162 OTUs whose relative abundance (RA) was significantly (*p* < 0.05, *t*-test) altered in the rhizosphere and the endosphere, respectively (Fig. 1A and Supplementary Table S1D). Approximately equal numbers of these OTUs showed increased or decreased RA in both the endosphere and the rhizosphere. These OTUs belonged to 79 taxanomic families (Supplementary Table S1D). These results indicate that flavonoids broadly contribute to the diversity of the Arabidopsis root microbiome. At the genus level, the *pap1-D* mutation altered the RA of 5 and 10 genus in the rhizosphere and the endosphere, respectively (Fig. 1B). *Aeromonas* was the only genus that showed increased RA in both compartments (Supplementary Table S1E). Similarly, at the level of families, only *Aeromonadaceae* showed higher RA in *pap1-D* than Col-0 in both the rhizosphere and the endosphere (Fig. 1C and Fig. S2B and Supplementary Table S1F). In this sense, flavonoids appear to preferentially attract *Aeromonadaceae* in the root microbiome under the assayed conditions.

### The flavonone naringenin induces chemotaxis in *Aeromonas* sp. H1 with enhanced bacteria motility

**Fig. 2:**
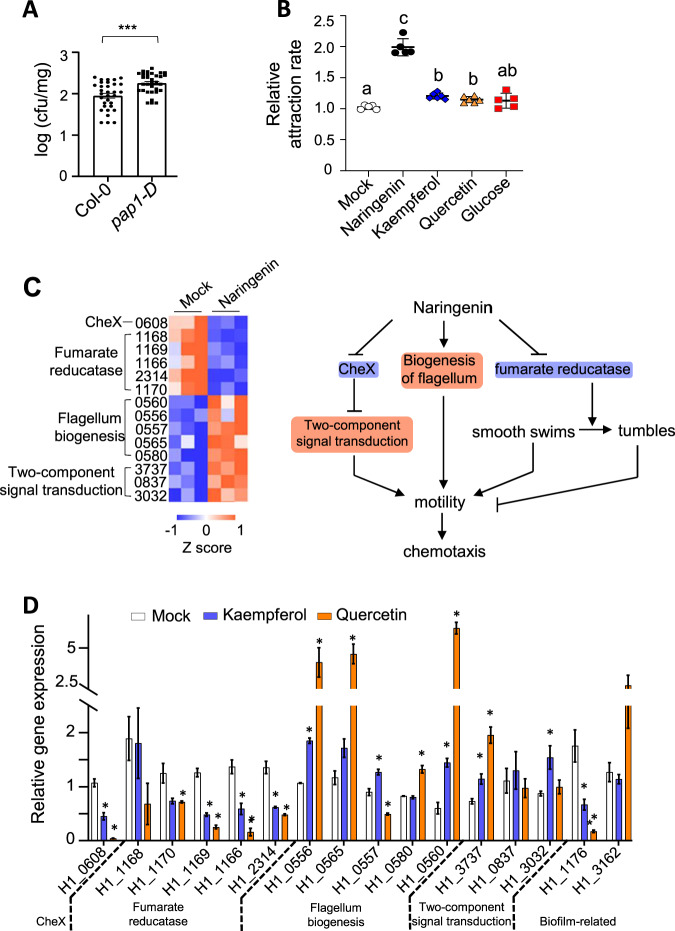
Naringenin enhances Arabidopsis interaction with *Aeromonas* sp. H1 through transcriptional enhancement of bacteria motility and colonization. **A** *Aeromonas* sp. H1 showed increased colonization on *pap1-D* compared to Col-0. Colony forming units (CFUs) were quantified per fresh weight of roots. Mean ± SE, *n* = 32 biological replicates. Three independent experiments were performed with similar results. *** indicates Student’s *t* test *p* ≤ 0.001. **B** *Aeromonas* sp. H1 showed chemotaxis to naringenin, quercetin and kaempferol. Chemotaxis-assayed cells with or without 100 μM chemo-attractants were collected and were lysed and quantified using CyQUANT GR fluorescent dye. Mean ± SE, *n* = 5 biological replicates. Three independent experiments were performed with similar results. Letters denote groups with significant statistical difference assessed by one-way ANOVA with Benjiamini multiple comparison. **C** RNA-seq analysis identified a group of chemotaxis-related DEGs (FDR ≤ 0.05, *n* = 3 biological replicates) in naringenin-treated *Aeromonas* sp. H1 (left panel), indicating transcriptional enhancement of bacterial motility via multiple mechanisms (right panel). **D** Quantitative RT-PCR results; mean ± SE, *n* = 3 technical replicates. Three independent experiments showed similar results. * indicates Student’s *t* test *p* ≤ 0.05.

We screened the cultivable soil bacteria and isolated an *Aeromonas* sp. numbered as Strain H1, whose 16S rRNA gene showed 100% homology to a flavonoid-responsive OTU (Fig. S3). The *Aeromonas* sp. H1 showed increased colonization on *pap1-D* compared to Col-0 (Fig. 2A). Chemotaxis assays demonstrated that H1 was attracted by naringenin (Fig. 2B), which is a key intermediate molecule in flavonoid biosynthesis and is a flavanone common in legumes and non-legumes (Fig. S4A).

To further understand how these colonization-related processes were regulated by flavonoids, we sequenced Strain H1 genome and subsequently examined Strain H1 transcriptome in response to naringenin. A total of 602 differentially expressed genes (DEGs) (fold-changes ≥2; FDR ≤ 0.05) were identified in naringenin-treated H1 (Supplementary Table S2). Categorization of the up- and down-regulated DEGs indicated that naringenin broadly influences many cellular processes in Strain H1 (Fig. S4B). Importantly, a group of 14 DEGs were identified as related to bacterial chemotaxis (Fig. 2C and Fig. S5A–C). These chemotaxis DEGs include eight up-regulated genes, which encode flagellar proteins (3 DEGs), flagellar biosynthetic proteins (2 DEGs), and signal transduction or receptor proteins (3 DEGs), indicating that naringenin enhances flagellar motor biogenesis in Strain H1. The chemotaxis DEGs also include six down-regulated genes, which encode the phosphatase CheX (1 DEG), a regulatory protein of fumarate reduction (1 DEG), and the subunits of fumarate reducatase (4 DEGs). CheX dephosphorylates and inhibits the response regulators CheY-P that mediate the signaling for chemotaxis, thereby imposing a negative regulation on bacterial motility [34]. Thus, down-regulation of CheX is consistent with up-regulation of the flagellar-related genes in enhancing Strain H1 motility. During chemotaxis, swimming modes of the bacterium is determined by the direction of flagellar motor, with counterclockwise rotation pushing the bacterium forward and clockwise rotation causing cell reorientation [34]. Fumarate assists the flagellar motor, through the fumarate reductase complex that binds to the motor proteins, to switch from counterclockwise to clockwise rotation, thereby turning smooth swims into tumbles [35]. Thus, down-regulation of the fumarate reduction-related genes supports chemotaxis through enhancing bacterial motility. Together these results indicate that naringenin-triggered Strain H1 chemotaxis is supported by the enhancement of flagellum biogenesis as well as the attenuation of fumarate-dependent suppression of bacterial motility.

Naringenin also induced gene expression of a F17 fimbrial protein (H1_1176) (Fig. S5D), which facilitate bacterial adherence to host cells [36], thus supporting the naringenin-induced biofilm production in Strain H1 (Fig. S5E). In contrast to naringenin, the same dose of glucose did not trigger either chemotaxis or the related gene regulation (Fig. 2B and Fig. S5A–D), although glucose also triggered biofilm production (Fig. S5E). To test whether the transcriptional regulation of naringenin-triggered chemotaxis is a common mechanism in different bacteria species, we examined *Bacillus megaterium* YC4-R4, of which the genome was previously sequenced [37]. The same treatment with naringenin did not trigger either chemotaxis or the Strain H1-related gene regulation in YC4-R4 (Fig. S5F, G), whose genome contains genes homologous to Strain H1’s CheX and three of the flagellum-related DEGs but not fumarate reductase [37]. Yet, YC4-R4 displayed decreased biofilm production in response to naringenin (Fig. S5H), indicating that naringenin elicited different molecular responses in the two bacteria species.

Naringenin is the biosynthesis precursor as well as the microbial degradation intermediate of the flavonols kaemferol and quercetin found in soil [38]. Similar to naringenin, these two flavonols triggered chemotaxis, transcriptionally regulated fumarate reductase and flagellum proteins, and increased biofilm production in Strain H1 (Fig. 2B, D and Fig. S5I). Therefore, flavonoids enhance plant interaction with *Aeromonas* sp. H1 through transcriptional regulation of bacteria motility and colonization. These results, together with the comparison between naringenin and glucose as well as the comparison between Strain H1 and YC4-R4, collectively suggest that the unique transcriptional regulation of bacterial motility likely explains the preferential attraction of *Aeromonadaceae* in the flavonoid-responsive microbiome, albeit this inference is limited as only two species with known genomes were tested.

### *Aeromonas* sp. H1 requires flavonoids for enhancing plant dehydration resistance

**Fig. 3:**
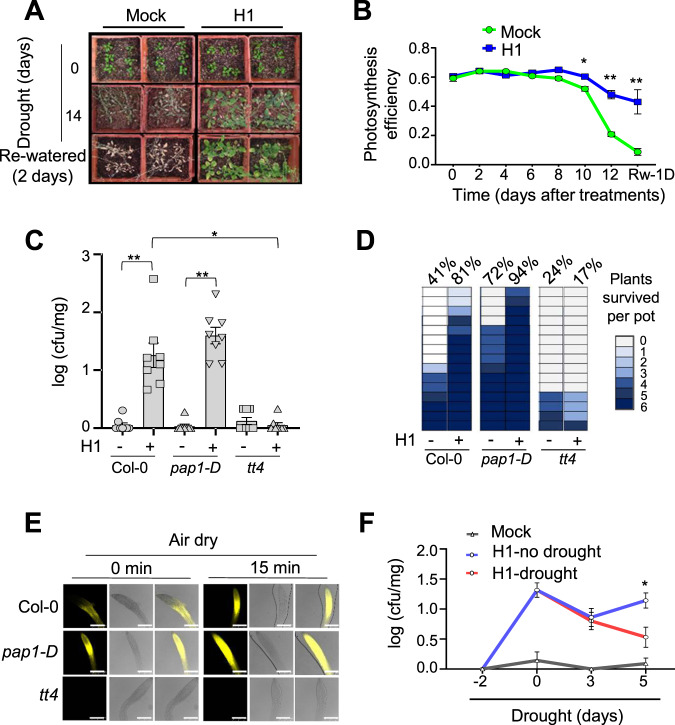
*Aeromonas* sp. H1-enhanced plant dehydration resistance requires flavonoid production but not in a cry-for-help manner. **A** Arabidopsis inoculated with *Aeromonas* sp. H1 showed increased resistance to dehydration stress. The plants were under the dehydration condition for the indicated time. **B** Arabidopsis inoculated with *Aeromonas* sp. H1 showed less reductions in photosynthesis efficiency compared to the control plants. For each data point, mean ± SE, *n* = 10 biological replicates. Three independent experiments showed similar results. * and ** indicates Student’s *t* test *p* ≤ 0.05 and *p* ≤ 0.01, respectively. RW-1D, 1 day after being re-watered. **C** *Aeromonas* sp. H1 failed to colonize the Arabidopsis mutant *tt4*, which is defective in flavonoid biosynthesis. Bacteria colonization was measured at 3 days after the dehydration treatment. Each data point represents the average colonization rate of six biological replicates in an independent experiment (*n* = 8), the bars show values of mean ± SE of the eight data points. * and ** indicates Student’s *t* test *p* ≤ 0.05 and *p* ≤ 0.01, respectively. Three independent experiments showed similar results. **D** *Aeromonas* sp. H1 failed to increase dehydration resistance in *tt4* with 13 days of the dehydration treatment. Data presents 15 biological replicates (soil pots), each containing six plants. The total survival rates (%) were shown above each stacked bar. The varying survival patterns in each pot were shown by the indicated color scale. Three independent experiments showed similar results. **E** Dehydration by air drying quickly increased flavonoid levels in Arabidopsis roots, as measured by fluorescence microscopy with diphenylboric acid 2-amino ethyl ester (DPBA) staining. Five independent experiments (*n* ≥ 40) showed similar patterns. White bars indicate 250 μm. **F** Dehydration stress did not lead to increased colonization of *Aeromonas* sp. H1 on Arabidopsis plants. Mean ± SE, *n* ≥ 6 biological replicates. Three independent experiments showed similar results. * indicates Student’s *t* test *p* ≤ 0.05.

To investigate the potential of Strain H1 in affecting plant growth and stress resistance, we next performed standard assays to evaluate its capacity in producing extracellular metabolites known as generally beneficial to plants. The characterization of H1 revealed its multiple plant-beneficial traits [39–41] (Fig. S6A–F), including production of the phytohormone auxin, production of ACC (1-aminocyclopropane-1-carboxylate) deaminase, phosphate solubilization, siderophore production, and decomposition of extracellular hydrogen peroxide (H_2_O_2_), but not polyamine production. We then examined H1’s effects on plant resistance to dehydration stress by withholding watering under 28 °C that is a hot temperature to *A. thaliana* [42], in order to mimic a natural hot and dry condition. Soil inoculation of H1 increased dehydration resistance in *A. thaliana*, as shown by the substantially improved plant survival and photosynthesis efficiency (Fig. 3A, B). H1-induced resistance to dehydration was also observed in other plant species such as rice and coriander (Fig. S6G, H). The Arabidopsis *tt4* mutant is defective in flavonoid production [43]. H1 failed to colonize the dehydration-stressed *tt4* and consequently showed no bacteria-enhanced dehydration resistance in *tt4* (Fig. 3C, D and Fig. S7A). Therefore, flavonoids are required for Strain H1-enhanced plant dehydration resistance.

### Bacterial enhancement of plant dehydration resistance does not rely on a sudden cry-for-help

Under the dehydration stress, the soil water contents quickly dropped from ~40% to <10% in 4 days (Fig. S7B), by which time the plants had not shown any reduction in photosynthesis efficiency (Fig. 3B), whereas the anthocyanin levels in both roots and shoots of the stressed plants were significantly increased within 3 days (Fig. S7C, D). Because anthocyanins are downstream products in the flavonoid biosynthesis pathway (Fig. S4A), these kinetic patterns indicated that the stress-induced flavonoid accumulation can be quick enough to occur before the stress-induced damages in photosynthesis. Indeed, Arabidopsis roots under dehydration stress increased flavonoid accumulation within 15 min (Fig. 3E). The relatively fast responses suggested that stress-induced flavonoids may mediate cry-for-help that results in the H1-increased plant resistance to dehydration. However, while H1 showed similar colonization rates on both the stressed and the control plants at 3 days-after-treatment (DAT), the dehydration stress unexpectedly reduced Strain H1 colonization at 5 DAT (Fig. 3F). These results indicate that, in addition to increasing flavonoid accumulation in plants, the dehydration stress also caused certain other effects that counteracted flavonoid-mediated attraction of H1. Nonetheless, flavonoids are required for H1 colonization to dehydration-stressed plants and for the bacteria-enhanced plant dehydration resistance (Fig. 3D, F). Thus, these results suggest that the flavonoid-dependent beneficial effects caused by H1 rely on pre-established association instead of a sudden cry-for-help under abiotic stress conditions. Similar to Strain H1, *Bacillus megaterium* TG1-E1 and *Bacillus amyloliquefaciens* GB03, which are two other plant-beneficial rhizobacteria capable of increasing plant dehydration resistance [19, 44], also showed dehydration-induced reductions in root colonization (Fig. S7E, F). These results collectively suggested that a sudden cry-for-help upon dehydration stress is unlikely effective in achieving bacteria-enhanced plant stress resistance, since the overall impact of dehydration stress on the binary plant-rhizobacteria association is negative.

### *Aeromonas* sp. H1 boosted dehydration-induced H_2_O_2_ in guard cells and stomatal closure

**Fig. 4:**
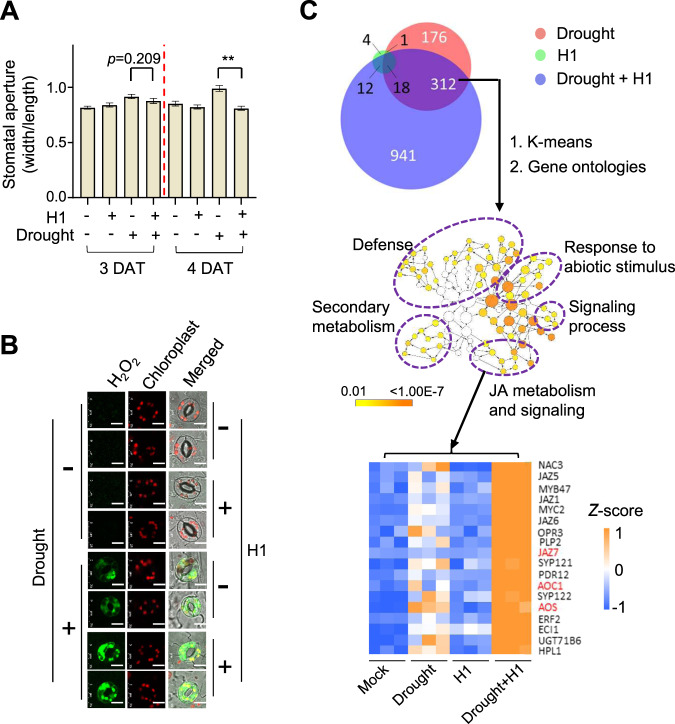
Synergistic responses to dehydration and *Aeromonas* sp. H1 resulted in increased efficacy of plant dehydration resistance. **A** *Aeromonas* sp. H1 induced stomatal closure in dehydration-stressed Arabidopsis. The plants were under the dehydration condition for the indicated time. Mean ± SE, *n* ≥ 29 stomata from four leaves for each sample. ** indicates Student’s *t* test *p* ≤ 0.01. Two independent experiments showed similar results. **B** *Aeromonas* sp. H1 increased ROS accumulation in the guard cells of dehydration-stressed Arabidopsis. The plants were grown at 28 °C with dehydration treatment for 3 days. ROS levels were indicated by fluorescent signals from the oxidation-sensitive CM-H_2_DCFDA. Two representative replicates of each sample (*n* ≥ 10) are shown. Three independent experiments showed similar results. White bars indicate 10 μm. **C** RNA-seq analysis of Arabidopsis transcriptome revealed plant’s synergistic responses to the dehydration stress and to *Aeromonas* sp. H1. DEGs numbers are indicated in the venn diagram on the top of the panel. The overlap DEGs were subjected to K-means clustering for dehydration-induced DEGs that were further induced by dehydration plus Strain H1 (see also Fig. S8D) and subsequently GO analysis to identify enriched biological processes (middle of the panel), which highlighted a group of JA-related DEGs including ACO1, AOS, and JAZ7 that are known to promote plant resistance to dehydration stress, as shown in the heat map at the bottom of the panel.

Under the dehydration stress, H1 helped the plants reduce water evaporation, as indicated by higher leaf temperatures and smaller stomatal aperture in the inoculated plants than the non-inoculated plants (Figs. 4A and S8A–C). Arabidopsis leaves respond to hot temperatures with increased stomatal aperture in order to decrease leaf temperature through water evaporation [45]. Consistently, the stressed plants initially showed increased stomatal aperture compared to the non-stressed plants at 3 and 4 DATs, whereas the stomatal aperture in the stressed plants dropped back to the levels similar to those in non-stressed plants at 5 and 6 DATs, indicating that the protection from dehydration eventually had a higher priority than keeping the leaf cool at a later stage. Strain H1 significantly reduced stomatal aperture at both the initial and the later stages in the stressed plants (Figs. 4A and S8C). Strain H1 also induced higher levels of H_2_O_2_, an important signal for stress-induced stomatal closure [46], in the guard cells of dehydration-stressed plants (Fig. 4B). In contrast, the plants without dehydration stress showed neither H_2_O_2_ accumulation nor stomatal closure in response to Strain H1 (Figs. 4A, B and S8C).

### Synergistic responses to dehydration and *Aeromonas* sp. H1 increased plant stress resistance

The transcriptome analysis at 3 DAT of dehydration further revealed a synergistic relation between plant responses to the dehydration stress and to H1; the former and the latter stimuli induced 507 and 35 DEGs (treated/control fold change >1.5, FDR < 0.05), respectively, whereas their combination resulted in 1283 DEGs (Fig. 4C and Supplementary Table S3). Since H1 increases plant dehydration resistance, it can be deduced that H1 enhances the efficacy of plant responses to the dehydration stress. Consistent with this notion, K-means clustering identified 145 dehydration-induced DEGs that were further induced by dehydration plus H1 (Figs. 4C and Fig. S8D and Supplementary Table S3), meanwhile Gene Ontology analysis revealed that these DEGs are enriched with several stress response-related biological functions, including secondary metabolism, defense, responses to abiotic stimulus, signaling process, and jasmonic acid (JA) metabolism and signaling (Fig. 4C and Supplementary Table S3). Dehydration stress increases the production of the JA precursor 12-oxo-phytodienoic acid (12-OPDA), which promotes stomatal closure in several plant species including Arabidopsis [47]. Consistent with Strain H1-promoted stomatal closure in the dehydration-stressed plants (Fig. 4A and Fig. S8C), Strain H1 further increased the dehydration-triggered gene induction of AOC1 and AOS (Fig. 4C and Supplementary Table S3), which are two key enzymes in 12-OPDA biosynthesis [47]. The JA-related DEGs also include JAZ7, whose transgenic overexpression resulted in enhanced dehydration resistance in *A. thaliana* [48]. Strain H1 further increased the dehydration-triggered gene induction of JAZ7 from 4 folds to 41 folds (Fig. 4C and Supplementary Table S3). Therefore, the Strain H1-increased efficacy of plant dehydration resistance can be attributed to the synergistic regulation of these JA-related processes, in addition to the production of ACC deaminase (Fig. S6B), which is known to contribute to bacteria-enhanced plant dehydration resistance [49].

## Discussion

In this study, we performed a metabolite-dependent microbiome profiling and accordingly identified *Aeromonas* sp. H1 that is attracted by flavonoids. We demonstrated that Strain H1 is a beneficial bacterial strain capable of enhancing dehydration resistance in various plant species, followed by the elucidation of how Strain H1 is attracted by flavonoids, as well as how Strain H1 enhances plant dehydration resistance in a way that requires flavonoids but not through a sudden cry-for-help. These findings reveal a key role of flavonoids, and the underlying mechanism, in mediating plant-microbiome interactions including the bacteria-enhanced plant dehydration resistance.

We showed that flavonoids broadly contribute to the diversity of the Arabidopsis root microbiome with a highlight on the attraction of *Aeromonadaceae*. The application of a genetic mutant with elevated flavonoid production allowed for the identification of microbiome members that were directly correlated to the altered flavonoid levels. An alternative starting strategy could be examining the root microbiome of dehydration-stressed plants, albeit this would require additional work to validate a causal relation between the alterations in the bacteria abundance and the alterations in the flavonoid levels. The same concern similarly exists for the strategy of starting from comparisons between plant mutants having different drought responses. In this sense, the strategy applied in this study is preferred for the efforts in searching for beneficial microbes whose effects are mediated through certain plant metabolites. While Strain H1 positively contributes to plant resistance to dehydration, it is worth to note that the bacterial strains less associated with the roots may also play roles in stress responses including drought.

In this study, we compared the root microbiome of *pap1-D* with that of the wild type plants, aiming to identify beneficial bacteria whose root colonization rates are positively correlated with the elevated plant production of flavonoids. The microbiome profiling highlighted the uniqueness of *Aeromonadaceae*, in that it is the only bacterial family that showed higher RA in *pap1-D* than Col-0 in both the rhizosphere and the endosphere; in addition, *Aeromonadaceae* showed not only greater fold changes but also greater statistical significance compared to the other families that showed significant alterations in *pap1-D* relative to Col-0. Therefore, we subsequently looked for cultivable *Aeromonadaceae* members from the same soil used for the microbiome study. Among the three isolated *Aeromonas* spp., Strain H1 displayed the most plant-beneficial traits in an initial screening; in addition, the 16S rRNA gene sequence of Strain H1 matches the *Aeromonas* OTU836780 that showed higher RA in *pap1-D* than Col-0 in both the rhizosphere and the endosphere. Therefore, Strain H1 was sent for genome sequencing and intensively investigated in the current study. In addition to matching OTU836780 in the old database GreenGenes, a 16S rRNA gene sequence of Strain H1 also showed 100% match to OTU2341 when the microbiome was later analyzed with the updated SILVA database v.138.1. It is worth to note that the other two cultivable *Aeromonas* spp. also showed significantly increased colonization rates in *pap1-D* compared to Col-0. Besides, these two *Aeromonas* spp. increased plant tolerance to high salinity stress in Arabidopsis and soybean, although they appeared to be ineffective in increasing plant drought resistance (data not shown). The rhizosphere is a reservoir of microbes including plant-beneficial bacteria. While this study highlighted the uniqueness of *Aeromonas* in showing higher RA in *pap1-D* than Col-0 in both the rhizosphere and the endosphere, it is worth to note that this does not imply that microbes enriched in only one of these two compartments should be excluded from the list of potential plant-beneficial microbes.

As shown by *Aeromonas* sp. H1, flavonoids induce chemotaxis via multiple mechanisms and are required for the bacteria-enhanced plant dehydration resistance. Interestingly, the root association of Strain H1 was impaired under the stress condition despite of the stress-induced flavonoid accumulation. The mechanism underlying the reduction in Strain H1 colonization remains unclear. One possibility is that the dehydration stress directly decreased the abundance of the bacteria. It is also possible that the stress caused alterations in certain unknown root exudate components that have strong impacts on Strain H1 colonization and/or survival. Nonetheless, the stress-induced increases in flavonoid levels likely contribute to the mutualistic association by alleviating the negative impacts caused by the stress on the pre-established root-H1 association. These observations suggested that a sudden cry-for-help upon the abiotic stress probably is not feasible; instead, the bacteria-induced stress resistance relies largely on the plant-bacteria association pre-established before a severe stress condition. This conclusion is based on the binary system consisting of H1 and the plant. Nonetheless, this finding suggests that timing is a critical factor for the efficacy of microbe-enhanced crop stress resistance, particularly given that beneficial soil microbes applied in agriculture are typically enriched in the rhizosphere similarly as in the binary system.

Bacteria-enhanced plant resistance to dehydration stress is frequently attributed to bacterial secretion of ACC deaminase or polyamines, which reduces plant ethylene levels or serve as cellular stress-protectants, respectively [8, 19]. In addition, some bacterial strains secrete exopolysaccharides that can improve soil aggregation and maintain soil moisture in the rhizosphere and thereby help plants survive under water deficit conditions [19]. *Aeromonas* sp. H1 possesses multiple plant-beneficial traits, which may directly or indirectly contribute to better plant growth under the dehydration stress. Plants in natural environments often simultaneously confront biotic and abiotic stressors or stimulators. Thus optimizing the integrative responses is critical for plant survival and wellness, whereas the underlying complex crosstalk has long been challenging to be disentangled. In contrast to those classical explanations for microbe-enhanced plant resistance to dehydration, the role and the mechanism of plant integrative responses are often vague. Our results revealed that simultaneous exposure to H1 and the dehydration stress resulted in synergistic responses, which strongly increased the efficacy of plant resistance to dehydration. Particularly, transcriptional regulation of the OPDA- or JA-related processes was highlighted in the strong synergistic effects underlying the bacteria-enhanced plant dehydration resistance. These findings present an example where plant integrative responses to the biotic and abiotic stimuli clearly play a key role in bacteria-enhanced plant dehydration resistance, albeit the molecular trigger of the synergistic responses remains to be identified.

## Supplementary information

Figure S1

Figure S2

Figure S3

Figure S4

Figure S5

Figure S6

Figure S7

Figure S8

Supplementary figures and tables legends

Supplementary Table S1 microbiota -SILA138.1

Supplementary Table S2 Strain H1 RNA-seq

Supplementary Table S3 plant RNA-seq

Supplementary Table S4 primer lists

## Data Availability

The 16S rRNA gene sequencing data are available in the NCBI SRA under BioProject PRJNA764534. The raw data of *Aeromonas* sp. H1 RNA-seq is available in the NCBI GEO with the accession number GSE184873 (token: stknkeqattsnlsl). The raw data of Arabidopsis RNA-seq are available in the NCBI GEO with the accession number GSE184872 (token: mrulcquslvczxgh).
