# Correction to: Flavonoid-attracted *Aeromonas* sp. from the Arabidopsis root microbiome enhances plant dehydration resistance

**DOI:** 10.1038/s41396-022-01309-5

**Published:** 2022-08-25

**Authors:** Danxia He, Sunil K. Singh, Li Peng, Richa Kaushal, Juan I. Vílchez, Chuyang Shao, Xiaoxuan Wu, Shuai Zheng, Rafael J. L. Morcillo, Paul W. Paré, Huiming Zhang

**Affiliations:** 1 Shanghai Center for Plant Stress Biology, Center for Excellence in Molecular Plant Sciences, Chinese Academy of Sciences, Shanghai, 201602 China; 2 University of Chinese Academy of Sciences, 100049 Beijing, China; 3 Department of Chemistry and Biochemistry, Texas Tech University, Lubbock, TX 79409 USA; 4 Present Address: Department of Applied Sciences and Biotechnology, Shoolini University, Solan, India; 5 Present Address: Instituto de Tecnologia Química e Biológica (ITQB), Oeiras, Lisbon, Portugal; 6 Present Address: Instituto de Hortofruticultura Subtropical y Mediterránea “La Mayora” (IHSM-UMA-CSIC), Málaga, Spain

**Keywords:** Applied microbiology, Plant sciences

Correction to: *The ISME Journal* 10.1038/s41396-022-01288-7, published online 16 July 2022

In the initial online version of this article, Figure 4B was meant to show two replicates of each sample, but the two replicates of the sample “H1-treated, non-stressed” (rows #3 and #4) were accidentally showing one replicate. This flaw, with all the source data, was reported to the journal immediately after we were aware of it. Even though this flaw does not affect any of the conclusions in the article, we sincerely apologize for this oversight. Figure 4B has been corrected.

The corrected Fig. 4B is given below:
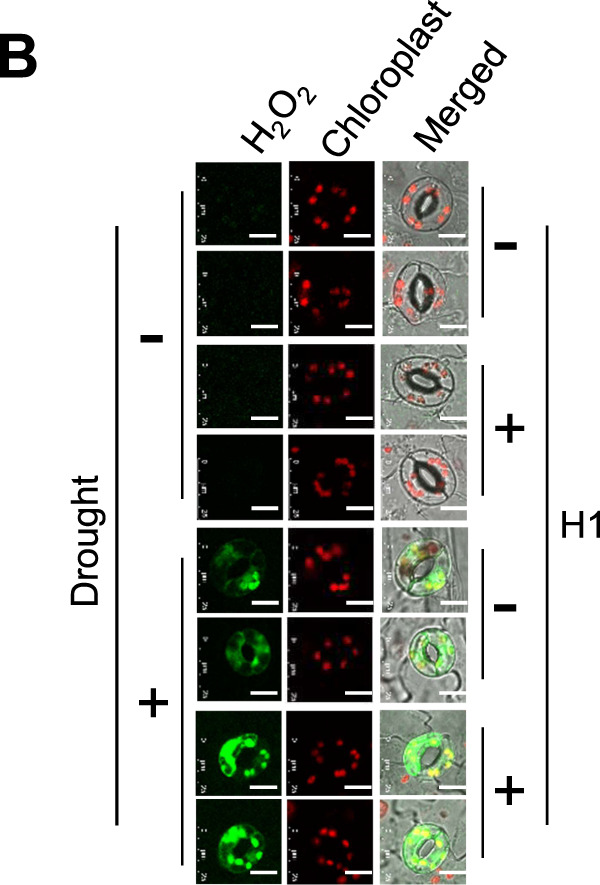


Fig. 4 Synergistic responses to dehydration and *Aeromonas* sp. H1 resulted in increased efficacy of plant dehydration resistance.

**B** *Aeromonas* sp. H1 increased ROS accumulation in the guard cells of dehydration-stressed Arabidopsis.

The plants were grown at 28 °C with dehydration treatment for 3 days. ROS levels were indicated by fluorescent signals from the oxidation-sensitive CM-H_2_DCFDA. Two representative replicates of each sample (*n* ≥ 10) are shown. Three independent experiments showed similar results. White bars indicate 10 μm.

The original article has been corrected.

