# Supplementary figures and images for: Flavonoid-attracted Aeromonas sp. from the Arabidopsis root microbiome enhances plant dehydration resistance
Source: ISME J. 2022 Jul 16;16(11):2622–32. doi: 10.1038/s41396-022-01288-7 (PMC9561528; doi:10.1038/s41396-022-01288-7)

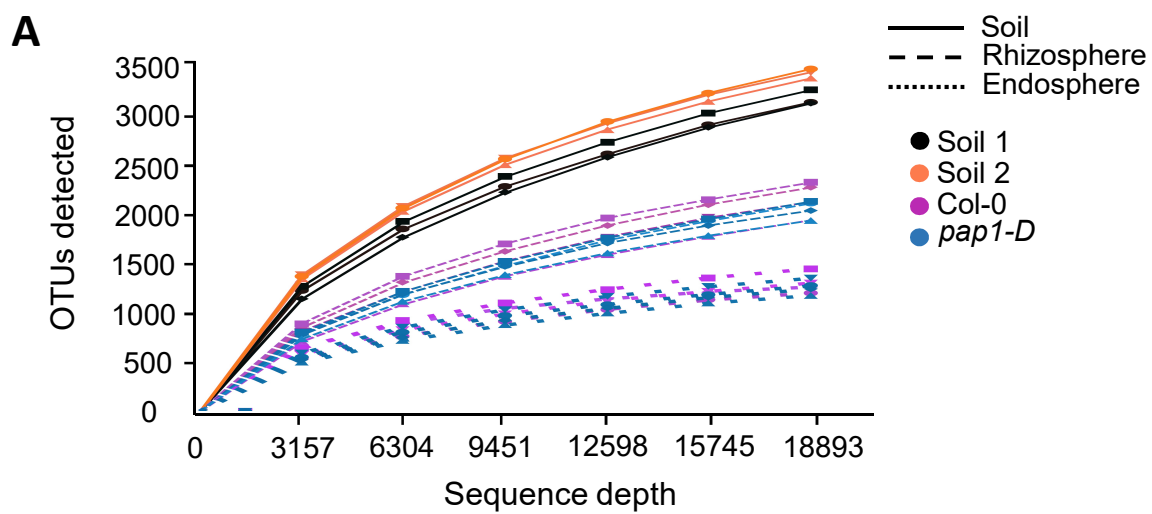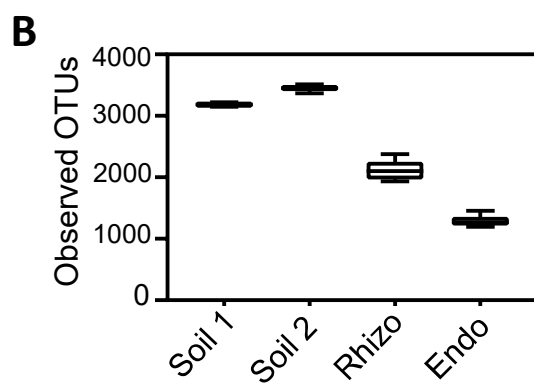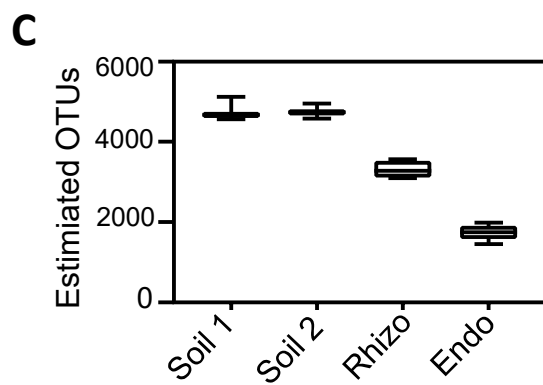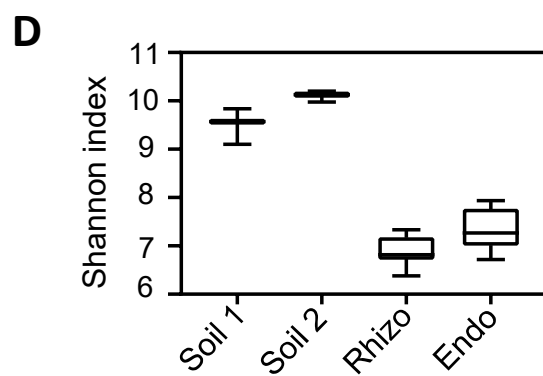

Supplement: Supplementary file 1 — Figure S1 [file 41396_2022_1288_MOESM1_ESM.pdf]

**A**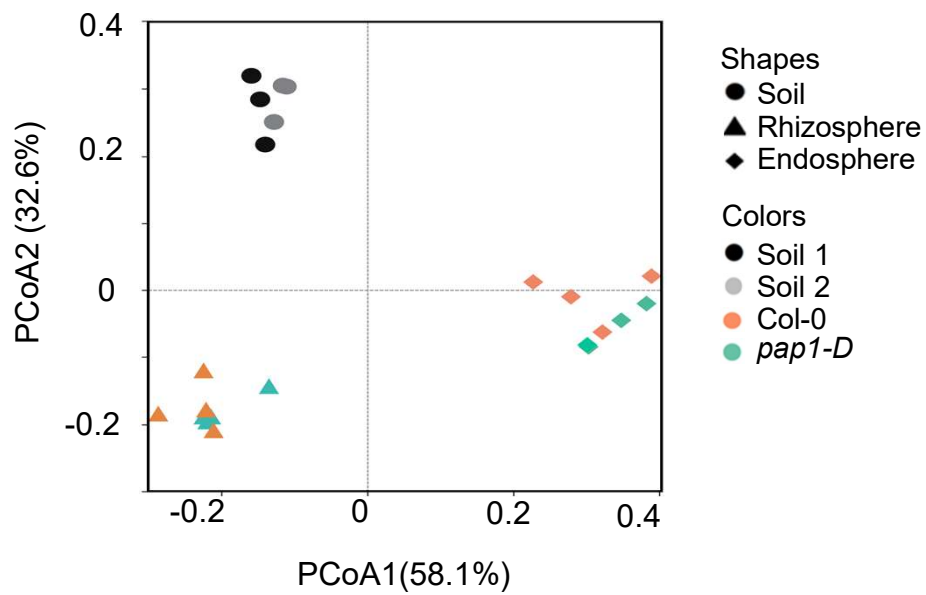**B**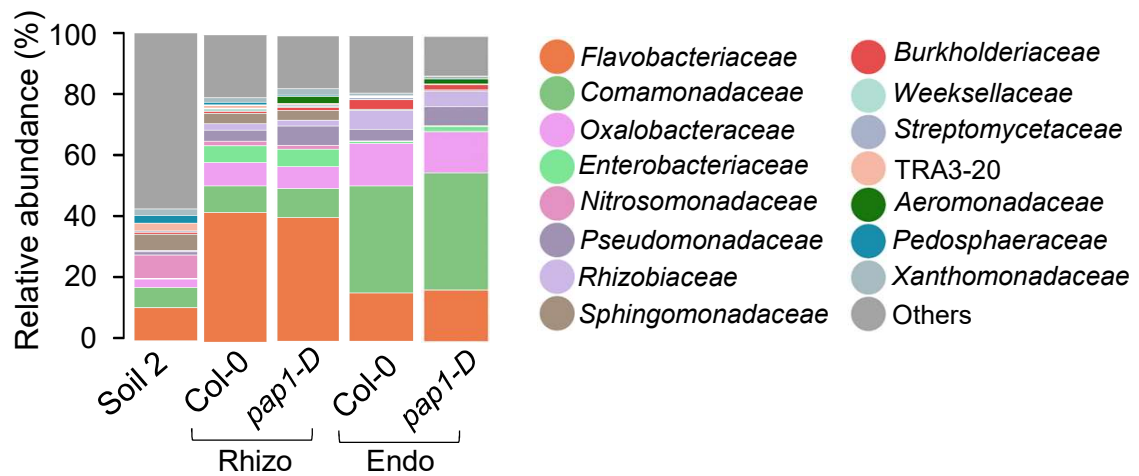

Supplement: Supplementary file 2 — Figure S2 [file 41396_2022_1288_MOESM2_ESM.pdf]

**A**

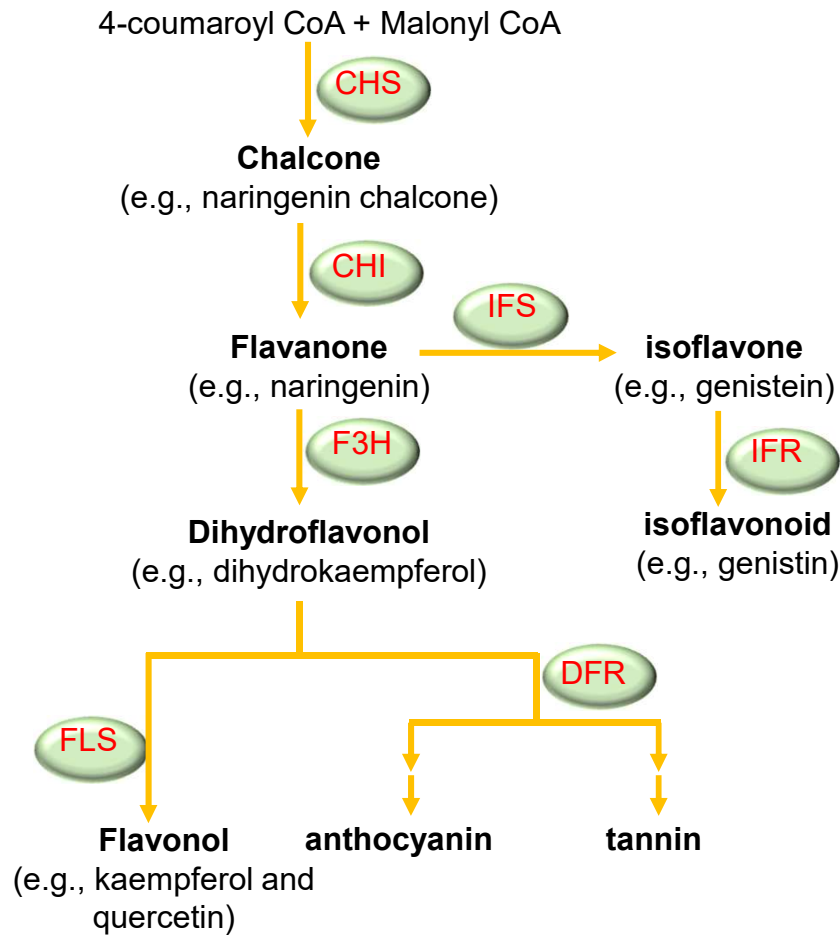

**B**

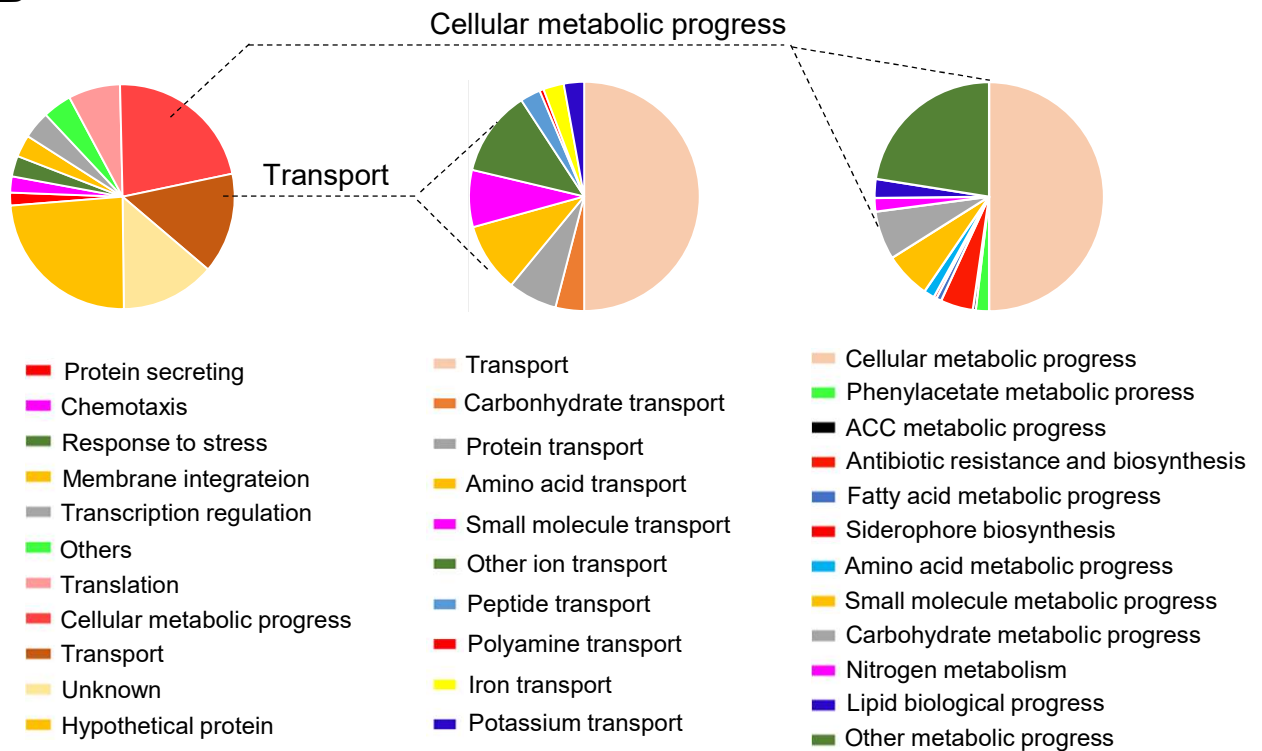

Supplement: Supplementary file 4 — Figure S4 [file 41396_2022_1288_MOESM4_ESM.pdf]

**A**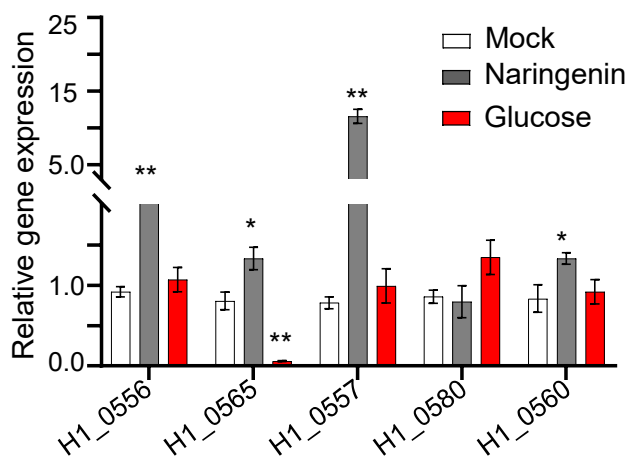**C**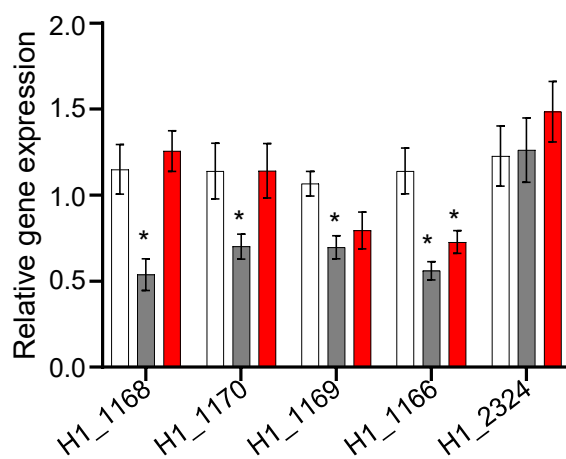**B**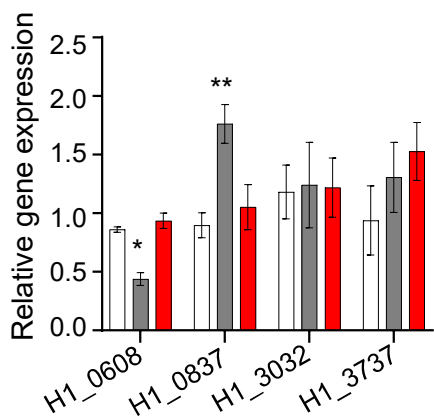**D**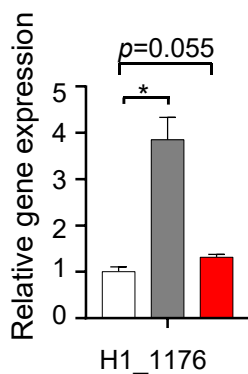**E**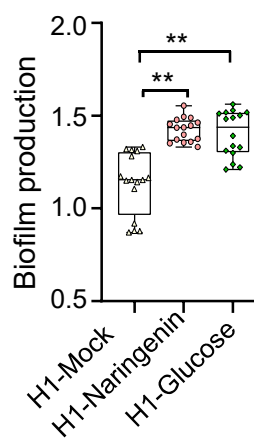**F**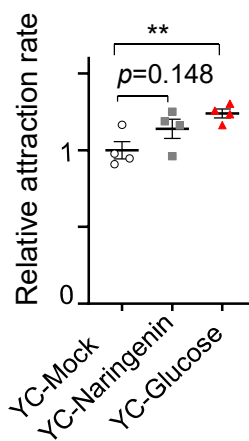**G**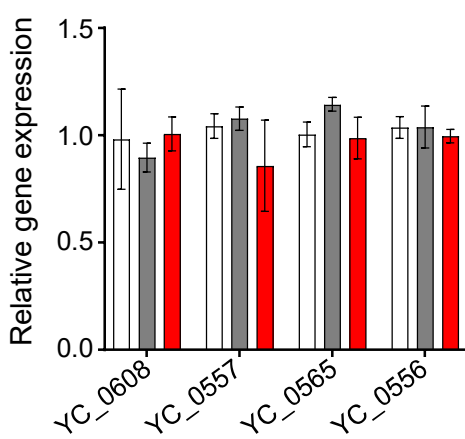**H**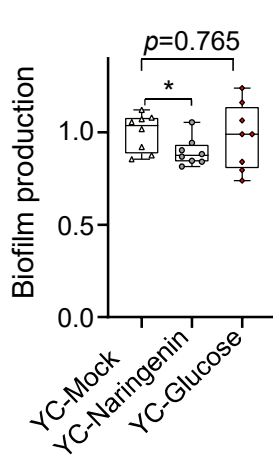**I**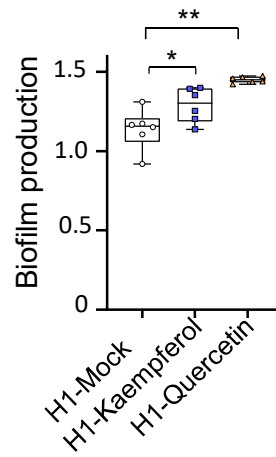

Supplement: Supplementary file 5 — Figure S5 [file 41396_2022_1288_MOESM5_ESM.pdf]

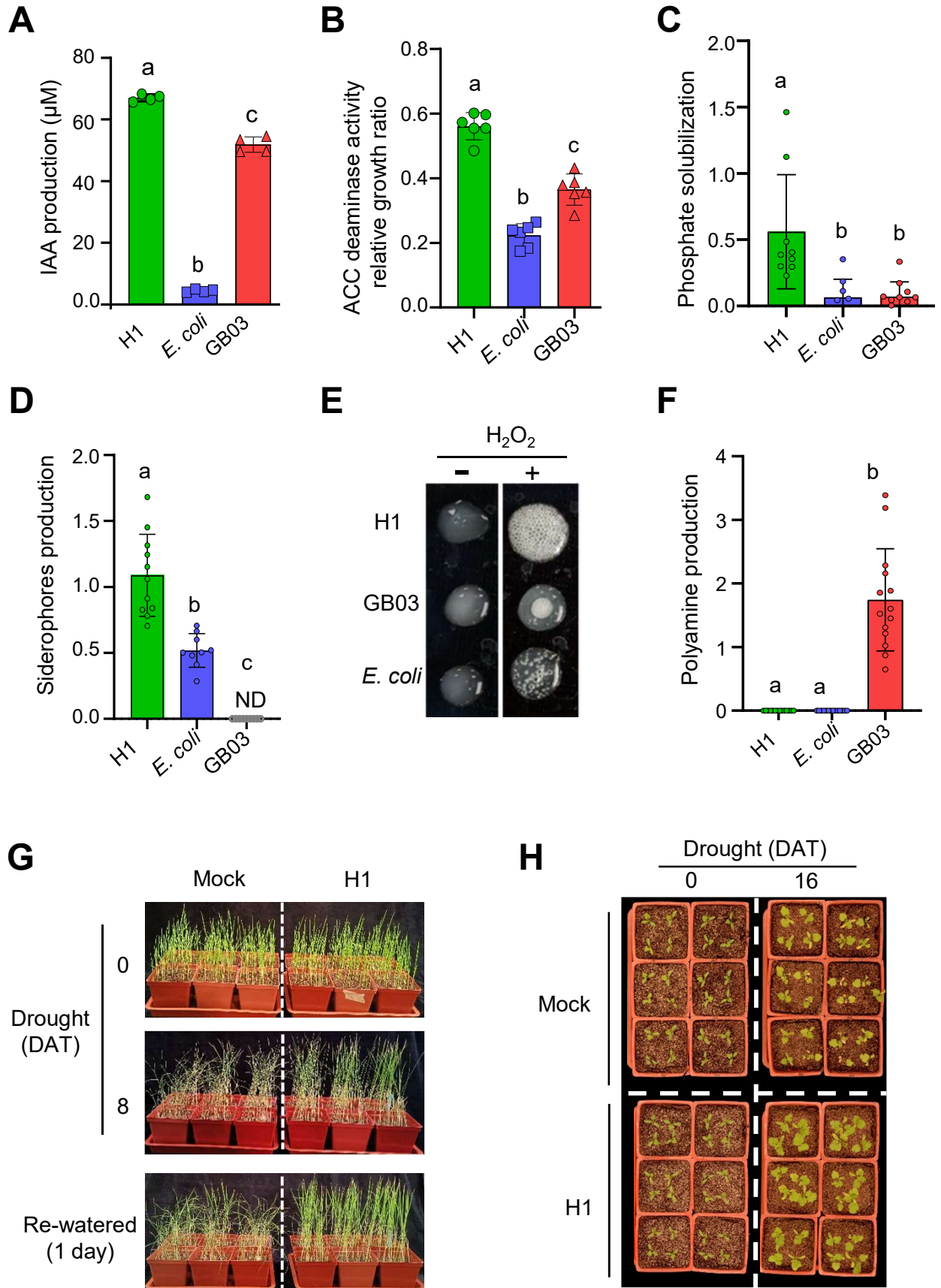

Supplement: Supplementary file 6 — Figure S6 [file 41396_2022_1288_MOESM6_ESM.pdf]

**A**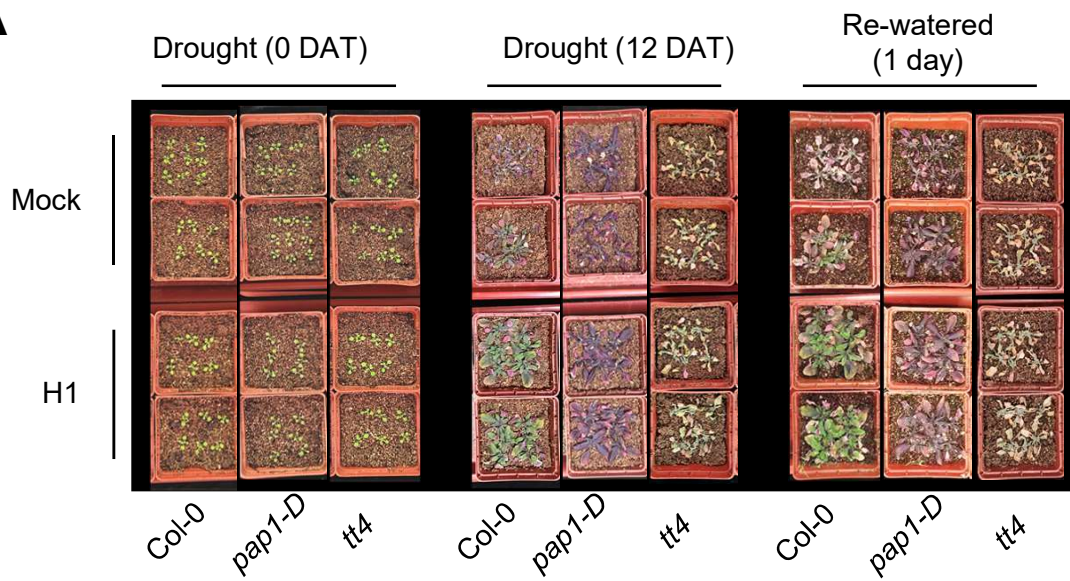**B**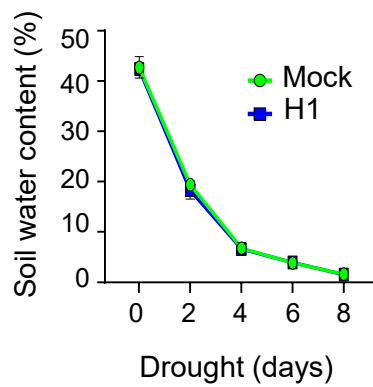**C**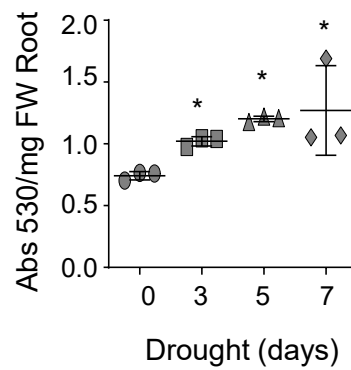**D**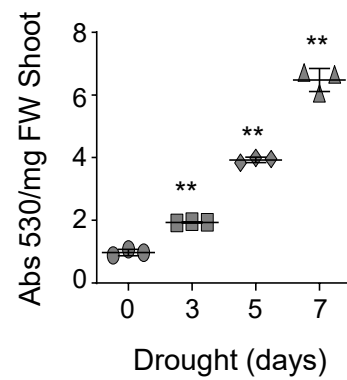**E**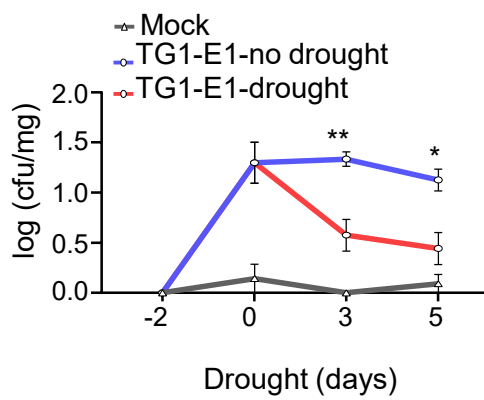**F**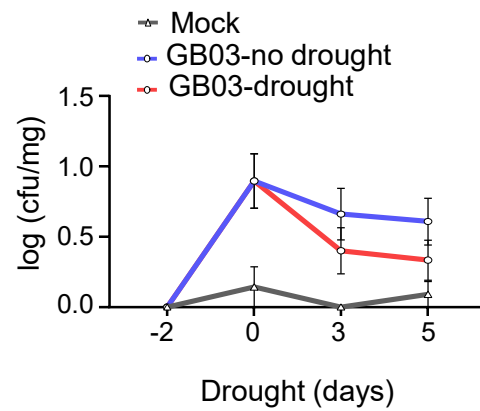

Supplement: Supplementary file 7 — Figure S7 [file 41396_2022_1288_MOESM7_ESM.pdf]

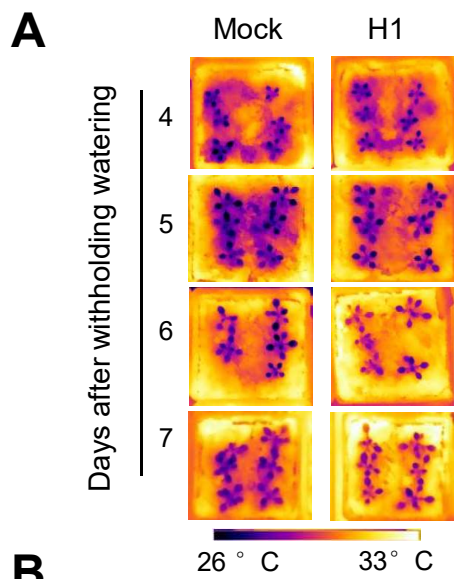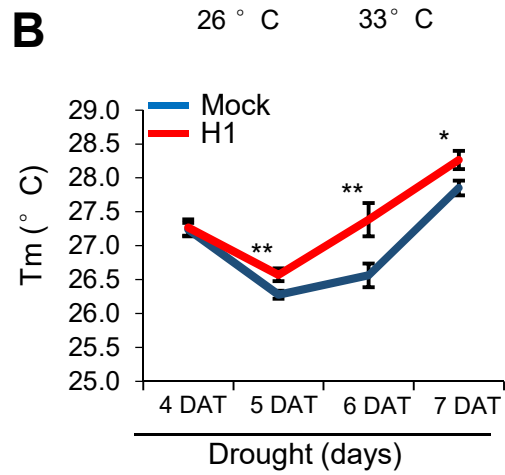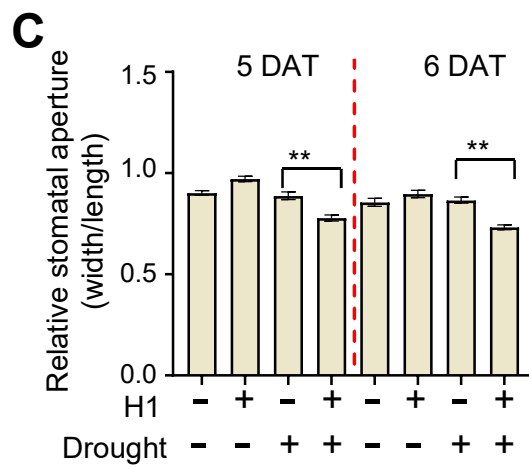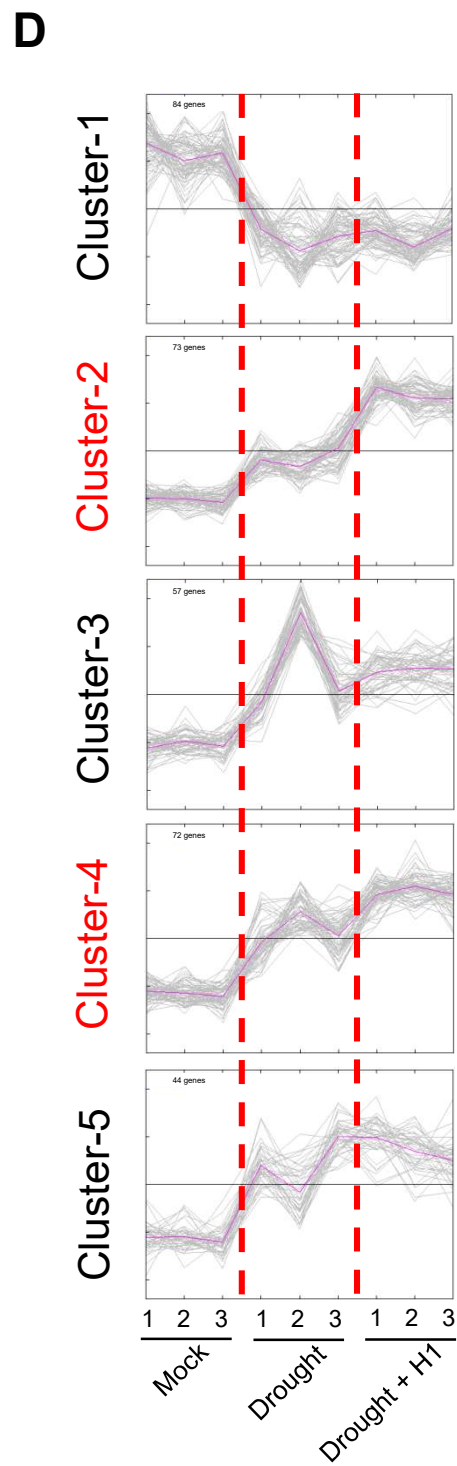

Supplement: Supplementary file 8 — Figure S8 [file 41396_2022_1288_MOESM8_ESM.pdf]
