# Supplementary material for: Flavonoid-attracted Aeromonas sp. from the Arabidopsis root microbiome enhances plant dehydration resistance
Source: ISME J. 2022 Jul 16;16(11):2622–32. doi: 10.1038/s41396-022-01288-7 (PMC9561528; doi:10.1038/s41396-022-01288-7)
Supplement: Supplementary file 3 — Figure S3 [file 41396_2022_1288_MOESM3_ESM.pdf]

```
839197          ---------------GTAGTCCACGCCGTAAACGATGTCGATTTGGAGGCTGTGTCCTTG    45
835322          ---------------GTAGTCCACGCCGTAAACGATGTCGATTTGGAGGCTGTGTCCTTG    45
836780          ---------------GTAGTCCACGCCGTAAACGATGTCGATTTGGAGGCTGTGTCCTTG    45
H1_16S:         GGATTAGATACCCTGGTAGTCCACGCCGTAAACGATGTCGATTTGGAGGCTGTGTCCTTG    840

839197          AGACGTGGCTTCCGGAGCTAACGCGTTAAATCGACCGCCTGGGGAGTACGGCCGCAAGGT   105
835322          AGACGTGGCTTCCGGAGCTAACGCGTTAAATCGACCGCCTGGGGAGTACGGCCGCAAGGT   105
836780          AGACGTGGCTTCCGGAGCTAACGCGTTAAATCGACCGCCTGGGGAGTACGGCCGCAAGGT   105
H1_16S:         AGACGTGGCTTCCGGAGCTAACGCGTTAAATCGACCGCCTGGGGAGTACGGCCGCAAGGT   900

839197          T AAAACTCAAATGAATTGACGGGGGCCCGCACAAGCGGTGGAGCATGTGGTTTAATTCGA  165
835322          T AAAACTCAAATGAATTGACGGGGGCCCGCACAAGCGGTGGAGCATGTGGTTTAATTCGA  165
836780          T AAAACTCAAATGAATTGACGGGGGCCCGCACAAGCGGTGGAGCATGTGGTTTAATTCGA  165
H1_16S:         T AAAACTCAAATGAATTGACGGGGGCCCGCACAAGCGGTGGAGCATGTGGTTTAATTCGA  960

839197          TGCAACGCGAAGAACCTTACCTGGCCTTGACATGTCTGGAATCCTG TAGAGATACGGGAG  225
835322          TGCAACGCGAAGAACCTTACCTGGCCTTGACATGTCTGGAATCCTG  CAGAGATGTGGGAG  225
836780          TGCAACGCGAAGAACCTTACCTGGCCTTGACATGTCTGGAATCCTG  CAGAGATGCGGGAG  225
H1_16S:         TGCAACGCGAAGAACCTTACCTGGCCTTGACATGTCTGGAATCCTG  CAGAGATGCGGGAG  1020

839197          TGCCTTCGGGAATCAGAACACAGGTGCTGCATGGCTGTCGTCAGCTCGTGTCGTGAGATG    285
835322          TGCCTTCGGGAATCAGAACACAGGTGCTGCATGGCTGTCGTCAGCTCGTGTCGTGAGATG    285
836780          TGCCTTCGGGAATCAGAACACAGGTGCTGCATGGCTGTCGTCAGCTCGTGTCGTGAGATG    285
H1_16S:         TGCCTTCGGGAATCAGAACACAGGTGCTGCATGGCTGTCGTCAGCTCGTGTCGTGAGATG    1080

839197          TTGGGTTAAGTCCCGCAACGAGCGCAACCCCTGTCCTTTGTTGCCAGCACGTAATGGTGG    345
835322          TTGGGTTAAGTCCCGCAACGAGCGCAACCCCTGTCCTTTGTTGCCAGCACGTAATGGTGG    345
836780          TTGGGTTAAGTCCCGCAACGAGCGCAACCCCTGTCCTTTGTTGCCAGCACGTAATGGTGG    345
H1_16S:         TTGGGTTAAGTCCCGCAACGAGCGCAACCCCTGTCCTTTGTTGCCAGCACGTAATGGTGG    1140

839197          GAACTCAAGGGAGACTGCCGGTGATAAACCGGA---------------------------   378
835322          GAACTCAAGGGAGACTGCCGGTGATAAACCGGA---------------------------   378
836780          GAACTCAAGGGAGACTGCCGGTGATAAACCGGA---------------------------   378
H1_16S:         GAACTCAAGGGAGACTGCCGGTGATAAACCGGAGGAAGGTGGGGATGACGTCAAGTCATC 1200
```
