# Supplementary material for: Flavonoid-attracted Aeromonas sp. from the Arabidopsis root microbiome enhances plant dehydration resistance
Source: ISME J. 2022 Jul 16;16(11):2622–32. doi: 10.1038/s41396-022-01288-7 (PMC9561528; doi:10.1038/s41396-022-01288-7)
Supplement: Supplementary file 9 — Supplementary figures and tables legends [file 41396_2022_1288_MOESM9_ESM.docx]

**Supplemental Figure Legends**

**Fig. S1. Bacteria richness in different compartments reflects the selectivity of plants on root-associated microbes (Related to Figure 1). (A)** Rarefaction curves of each sample. **(B)** Numbers of observed OTUs in the different compartments. **(C)** Numbers of estimated OTUs based on the Chao1 estimator. **(D)** Shannon index of the microbial diversity. Samples were rarefied to 18893 reads prior to the analysis. Soil 1, initial bulk soil; Soil 2, final bulk soil; Rhizo, rhizosphere; Endo, endosphere。

**Fig. S2. The overall impacts of compartment and genotype on the root-associated microbes (Related to Figure 1). (A)** PCoA (Principal Coordinate Analysis) using weighted UniFrac metric indicates that the microbiome assembly is mainly determined by the compartments (soil, rhizosphere, and endosphere). The bacteria communities were investigated by 16S rRNA gene metagenomic sequencing of the extracted DNA. n ≥ 3 biological replicates. **(B)** Relative abundance (RA) of the bacteria families that were identified in the final bulk soil, rhizosphere, and endosphere compartments.

**Fig. S3. The 16S rRNA gene sequence of *Aeromonas* sp. H1 showed 100% match to the flavonoid-responsive OTU 836780 (Related to Figure 2).** Sequence alignments are shown for the 16S rRNA gene amplicons from H1 and the three flavonoid-responsive OTUs belonging to *Aeromonadacea*. Letters in red indicate unmatched nucleotides. The OTU836780 was annotated based on the database GreenGenes. When the 16S rRNA gene sequencing results were reanalyzed later with the updated SILVA database v.138.1, Strain H1 showed 100% match to the identified *Aeromonas* OTU2341.

**Fig. S4. Genome-wide profiling of *Aeromonas* sp. H1 gene expression in response to naringenin (Related to Figure 2). (A)** A schematic pathway of flavonoid biosynthesis. CHS, chalcone synthase; CHI, chalcone isomerase; IFS, isoflavone synthase; IFR, isoflavone reductase; F3H, flavonoid 3-hydroxylase; FLS, flavonol synthase; DFR, dihydroflavonol 4-reductase. **(B)** Categorization of the DEGs (differentially expressed genes, treated vs mock fold change ≥ 2, FDR ≤ 0.05) identified by RNA-seq of *Aeromonas* sp. H1 treated with 100 µM naringenin for 8 hr. The top two largest groups of DEGs (except for hypothetical and unknown proteins), cellular metabolic processes and transport, are further categorized.

**Fig. S5. Naringenin transcriptionally regulates bacteria motility and biofilm production in *Aeromonas* sp. H1 (Related to Figure 2).** Quantitative RT-PCR measurements of *Aeromonas* sp. H1 genes involved in flagellum biogenesis **(A)**, two-component signal transduction including the negative regulator CheX **(B)**, fumarate reduction **(C)** or the F17 fimbrial protein H1_1176 **(D)**. The bacteria were treated with 100 µM naringenin or glucose for 8 hr. Mean ± SE, n = 4 from 2 biological replicates. Three independent experiments showed similar results. * indicates Student’s *t*-test *p* ≤ 0.05. **(E)** Biofilm production of *Aeromonas* sp. H1 treated with 100 µM naringenin or glucose for 24 hr. Whiskers in the boxplot represent the min to max data range, the median is represented by the central horizontal line. The upper and lower limits of the box outline represent the first and third quartile. Two independent experiments showed similar results. ** indicates Student’s *t*-test *p* ≤ 0.01. **(F)** Chemotaxis measurements of *B. megaterium* YC4-R4. Chemotaxis-assayed cells with or without 100 μM chemo-attractants were collected and were lysed and quantified using CyQUANT GR fluorescent dye. Mean ± SE, n = 4 biological replicates. Three independent experiments were performed with similar results. ** indicates Student’s *t*-test *p* ≤ 0.01. **(G)** Quantitative RT-PCR measurements of *B. megaterium* YC4-R4 CheX gene and some genes homologous to the flagellum biogenesis-related DEGs identified in *Aeromonas* sp. H1. Mean ± SE, n = 4 from 2 biological replicates. **(H)** Biofilm production of YC4-R4 treated with 100 µM naringenin or glucose for 24 hr. * in the boxplot indicates Student’s *t*-test *p* ≤ 0.05; n=6 biological replicates. Two independent experiments showed similar results. **(I)** Biofilm production of *Aeromonas* sp. H1 treated with 100 µM kaempferol or quercetin for 24 hr. * in the boxplot indicates Student’s *t*-test *p* ≤ 0.05; n = 6 biological replicates. Two independent experiments showed similar results.

**Fig. S6. Characterization of *Aeromonas* sp. H1 as a plant-beneficial bacterial strain (Related to Figure 3).** Multiple plant-beneficial traits were assayed, including production of the phytohormone auxin (panel **A**; n = 4 biological replicates), production of ACC deaminase (**B**; n = 6), phosphate solubilization (**C**; n ≥ 9), siderophore production (**D**; n ≥ 9), decomposition of extracellular H_2_O_2_ (**E**; representative images, n = 6), and production of polyamines (**F**; n = 14). A known plant-beneficial bacterial strain (*B. amyloliquefaciens* GB03) and the *Escherichia coli* strain DH5α were examined in parallel for comparison with Strain H1. Two independent experiments showed similar results. Different letters above the bars (mean ± SE) indicate statistical difference determined by one-way ANOVA. **(G)** Rice seedlings inoculated with *Aeromonas* sp. H1 showed increased resistance to dehydration stress. Six biological replicates (pots) are shown. Three independent experiments showed similar results. **(H)** *Coriander sativum* inoculated with *Aeromonas* sp. H1 showed increased resistance to dehydration stress. Six biological replicates (pots) are shown. Three independent experiments showed similar results.

**Fig. S7. *Aeromonas* sp. H1-enhanced plant dehydration-resistance requires flavonoid production but not in a cry-for-help manner (Related to Figure 3). (A)** *Aeromonas* sp. H1 increased plant dehydration resistance in Col-0 and *pap1-D* but not *tt4*. Representative images are shown (n ≥ 5 biological replicates). Three independent experiments showed similar results. **(B)** The water contents of soil surrounding Arabidopsis plants with or without Strain H1 treatment under the dehydration condition. Mean ± SE, n = 6 biological replicates. Four independent experiments showed similar results. **(C)** The anthocyanin levels were induced in Arabidopsis roots under dehydration stress persisted. Mean ± SE, n = 3 biological replicates, each replicate containing 9 seedlings. **(D)** The anthocyanin levels in dehydration-stressed Arabidopsis shoots. Mean ± SE, n = 3 biological replicates, each consisting 9 seedlings. Two independent experiments showed similar results. * and ** indicates Student’s *t*-test *p* ≤ 0.05 and *p* ≤ 0.01, respectively. **(E, F)** The colonization rates of *B. megaterium* TG1-E1 and *B. amyloliquefaciens* GB03 on Arabidopsis roots with or without the dehydration treatment. Mean ± SE, n = 3 biological replicates, each consisting 6 seedlings. Three independent experiments showed similar results. * and ** indicates Student’s *t*-test *p* ≤ 0.05 and *p* ≤ 0.01, respectively.

**Fig. S8. *Aeromonas* sp. H1 increased plant dehydration resistance through regulation of stomatal closure and stress-responsive gene expression (Related to Figure 4). (A)** Infrared thermal imaging of leaf temperatures of Arabidopsis inoculated with or without Strain H1 under the dehydration condition. Representative images of one biological replicate were shown for each sample. **(B)** Quantification of leaf temperatures measured by infrared thermal imaging. Mean ± SE, n ≥ 24 from 6 biological replicates. * and ** indicates Student’s *t*-test *p* ≤ 0.05 and *p* ≤ 0.01, respectively. **(C)** *Aeromonas* sp. H1 induced stomatal closure in dehydration-stressed Arabidopsis. *dat*, days after treatments. Mean ± SE, n ≥ 29 stomata from 4 leaves for each sample. ** indicates Student’s *t*-test *p* ≤ 0.01. Two independent experiments showed similar results. **(D)** K-means clustering analysis of the 330 DEGs that were identified by RNA-seq as commonly induced by the dehydration stress and by dehydration plus Strain H1. The clusters 2 and 4 identified the dehydration-induced DEGs that were further induced by dehydration plus Strain H1. The numbers on the x-axis indicate biological replicates.

**Supplemental Table Legends**

**Supplementary Table S1. 16S rRNA gene sequencing results. (A)** Total OTUs (operational taxonomic units) detected by the 16S rRNA gene sequencing. **(B)** List of OTUs with the rarefied quality sequence cutoff at 18893. **(C)** All genus identified in the microbiome. **(D)** List of OTUs with significantly (*p* < 0.05) altered RA. **(E)** Differentially enriched genus between *pap1-D* and Col-0. **(F)** Differentially enriched bacteria families between *pap1-D* and Col-0. **(G)** Sample information.

**Supplementary Table S2. RNA-seq results of naringenin-treated *Aeromonas* sp. H1. (A)** Naringenin-triggered DEGs in H1 (fold change > 2 and FDR < 0.05). **(B)** Uniprot clustering of the DEGs based on biological fucntions. **(C)** DEGs in the cluster of “Transport”. **(D)** DEGs in the cluster of “Cellular metabolic progress”. **(E)** DEGs in the cluster of “Chemotaxis”.

**Supplementary Table S3. RNA-seq of Arabidopsis plants with and with the dehydration and/or H1 treatments.** **(A)** DEGs of dehydration vs mock. **(B)** DEGs of H1 vs mock. **(C)** DEGs of dehydration+H1 vs mock. **(D)** DEGs commonly induced by dehydration and by dehydration+H1. **(E)** K-means clustering of the DEGs commonly induced by dehydration and by dehydration+H1. **(F)** Gene Ontology (GO) analysis of the DEGs in cluster 2 and cluster 4. **(G)** JA-related DEGs identified by K-means clustering and GO analysis.

**Supplementary Table S4. Primers used for preparing the 16S rRNA gene library and for quantitative RT-PCR.** **(A)** Primers for preparing 16S rRNA gene library. **(B)** Primers for qRT-PCR.
